# Computational State Space Models for Activity and Intention Recognition. A Feasibility Study

**DOI:** 10.1371/journal.pone.0109381

**Published:** 2014-11-05

**Authors:** Frank Krüger, Martin Nyolt, Kristina Yordanova, Albert Hein, Thomas Kirste

**Affiliations:** Computer Science Institute, University of Rostock, Rostock, Germany; University of East Piedmont, Italy

## Abstract

**Background:**

Computational state space models (CSSMs) enable the knowledge-based construction of Bayesian filters for recognizing intentions and reconstructing activities of human protagonists in application domains such as smart environments, assisted living, or security. Computational, i. e., algorithmic, representations allow the construction of increasingly complex human behaviour models. However, the symbolic models used in CSSMs potentially suffer from combinatorial explosion, rendering inference intractable outside of the limited experimental settings investigated in present research. The objective of this study was to obtain data on the feasibility of CSSM-based inference in domains of realistic complexity.

**Methods:**

A typical instrumental activity of daily living was used as a trial scenario. As primary sensor modality, wearable inertial measurement units were employed. The results achievable by CSSM methods were evaluated by comparison with those obtained from established training-based methods (hidden Markov models, HMMs) using Wilcoxon signed rank tests. The influence of modeling factors on CSSM performance was analyzed via repeated measures analysis of variance.

**Results:**

The symbolic domain model was found to have more than 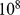 states, exceeding the complexity of models considered in previous research by at least three orders of magnitude. Nevertheless, if factors and procedures governing the inference process were suitably chosen, CSSMs outperformed HMMs. Specifically, inference methods used in previous studies (particle filters) were found to perform substantially inferior in comparison to a marginal filtering procedure.

**Conclusions:**

Our results suggest that the combinatorial explosion caused by rich CSSM models does not inevitably lead to intractable inference or inferior performance. This means that the potential benefits of CSSM models (knowledge-based model construction, model reusability, reduced need for training data) are available without performance penalty. However, our results also show that research on CSSMs needs to consider sufficiently complex domains in order to understand the effects of design decisions such as choice of heuristics or inference procedure on performance.

## Introduction

### 1.1 Motivation

Recently, a number of different approaches to representing the transition models of probabilistic state space models (SSMs) by computational means have been proposed as method for building intention recognition systems, from somewhat different research perspectives and conceptual backgrounds [1]–[5]. *Computational state space models* (CSSMs) are probabilistic models where the transition model of the underlying dynamic system can be described by any computable function using compact algorithmic representations. Objective of the study reported in this paper is to evaluate the applicability of CSSMs for the purpose of sequential state estimation in dynamic systems with very large state spaces and dense transition models. Such domains are difficult to handle with conventional methods relying on the explicit enumeration of states or paths, such as hidden Markov models (HMMs) and their various extensions [6], probabilistic context-free grammars [7], or (libraries of) (partially ordered) plans [8]. We specifically consider CSSMs for the objective of recognizing activities, goals, plans, and intentions of autonomous non-deterministic agents, such as human protagonists. These recognition tasks frequently arise in application domains like smart environments [9], [10], security and surveillance [11], man-machine-collaboration [2], and assistive systems [12], [13].

Researchers have chosen computational state space models for applications in activity and intention recognition for a range of different reasons. CSSMs have been considered because they allow to

- substitute training data by symbolic prior knowledge [3],
- replace explicit enumerations of possible action sequences by on-the-fly-synthesis of plans [5],
- enable the flexible introduction of additional state variables that allow inferences about, for instance, the cognitive state of a person [2],
- exchange observation models without affecting the system model in response to changing sensor setups [14].

While these properties seem desirable from the viewpoint of model development and model reusability, they come at a price: using computational symbolic descriptions, it is very easy to produce models with a very large state space. This is an immediate effect of the generalization and abstraction power of the computational representations: a model that considers not only an explicit enumeration of action sequences, but rather all sequences that achieve the same objective, will have a larger set of states. From the viewpoint of probabilistic inference, a large state space is first of all not an asset but a liability. Considering the bias-variance trade-off [15], a large state-space might produce a weaker performance (due to variance) than a smaller, potentially less flexible and more biased state space.

The use of CSSMs for activity and intention recognition has so far been investigated only in comparatively limited scenarios with small state spaces and only few activities to distinguish. It remains unclear, how well this approach scales to larger problems and in how far inference in large state spaces is tractable. Objective of the study presented in this paper is to answer this question. Our findings suggest that such problems can indeed be successfully tackled by CSSMs.

The further structure of this paper is as follows: as CSSMs are not yet widely established, a brief overview of the pertinent concepts of CSSMs is given in Sec. 1.2. A review of current empirical investigations of CSSMs for activity and intention recognition, including an assessment of the experimental scenarios, is contained in Sec. 1.3. In Sec. 1.4 we explain the experimental setting we used, the overall CSSM model structure used for activity reconstruction, as well as the inference algorithms. In Sec. 2.4.2 we present the quantitative data obtained from our experiments. A discussion of these results, our reasoning why we think these results justify the claim that CSSMs are capable to tackle real world scenarios, and the limitations of our study are given in Sec. 3.4. Notational conventions and abbreviations are summarized in Appendix S1, the remaining appendices contain supplemental material.

### 1.2 Computational state space models

This section provides a brief review of the pertinent notions of CSSMs from the perspective of intention recognition (IR) and activity recognition (AR). A detailed introduction and discussion is provided in Appendix S2.

We consider dynamic systems whose behavior can be formally captured by the notion of labeled transition systems (LTS). An LTS is a triple 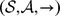 where 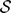 is a set of states, 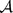 a set of action labels and 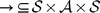 a labeled transition relation. It is easy to arrive at LTS where 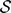 and 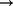 are infinite, even though 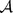 is finite – for instance by introducing states that represent counter values and actions that increment such counters. In the domain of intelligent assistive systems, protagonist activities such as setting a table by incrementally moving items from the kitchen to the dining room result in this counting behavior. Such LTS can not be represented by explicit enumeration of states and transitions, but if defined in a suitable algorithmic language (which we call “computational action language”), then also these LTS have a finite representation. As long as only a finite subset of states needs to be considered in a given intention recognition task, computations on such latently infinite systems remain feasible.

State space models (SSMs) [16] are a general class of probabilistic models that allow to infer the hidden state of an LTS (e. g. the current activity or location of items) given a sequence of observations (sensor readings). Let 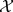 be some set of states, let 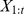 be a sequence of random variables with value domain 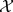. Furthermore, let 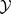 be a set of observations and 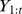 a sequence of random variables with value domain 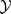. Then the joint distribution 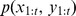 can be described by an SSM if it recursively (over time 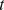) factorizes into a *transition model* 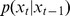 and an *observation model* 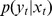, that is 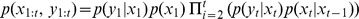. The underlying idea of *computational* state space models (CSSMs) is to use computational action languages for representing the transition distribution. This approach is interesting when the process under observation can be considered as performing some kind of sequential “computation”, including such phenomena as goal directed behavior of human protagonists.

We call 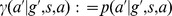 the action selection distribution, which models the non-deterministic behavior of protagonists in the case that multiple actions are applicable to a given situation. They encompass decision theoretic quantities, such as an “action's utility” in reaching the goal from the given state [17], as well as situation-based conflict resolution strategies such as “specificity” [14].

### 1.3 Feasibility of CSSMs for large scenarios: Current research

As discussed above, CSSMs provide a number of desirable properties. However, an analysis of current literature reviews on activity and intention recognition [18]–[20] shows that currently there is little empirical evidence for their applicability to detailed models of everyday situations. Current studies either use models that do not employ computational mechanisms, or they use scenarios of very limited detail and/or simulations. Below we summarize the results of this literature analysis.

#### 1.3.1 Survey criteria

We identified different features (*factors*) for assessing and comparing capabilities of the methods and complexities of experiments. Table 1 gives a brief explanation of the factors used for evaluation. The *F* factors represent the different properties provided by CSSMs, they show in how far the approach used in the respective study can be considered a CSSM. The *N* factors quantify the complexity of the experimental setting used for evaluation in the respective study. *N.subjects* indicates whether sensor data obtained from human subjects has been used, or simulations drawn from the model. The factor *N.State* gives a rough quantitative estimate of the model's detail level.

**Table 1:** Factors for analyzing empirical studies on activity recognition.

**F.latent.infty**	Method allows inference in latently infinite state spaces (typically employing a computational action language).
**F.plan.synth**	Plan synthesis is supported. Otherwise, the approach requires to create plan libraries by explicitly enumeration.
**F.duration**	Durative actions are supported. (This will significantly increase inference complexity, as the starting time for an action becomes another state variable, which has a large value space. See Appendix S4)
**F.action.sel**	Explicit mechanisms for modeling human action selection based on opportunistic and/or goal driven features are supported.
**F.probability**	Method provides (an approximation of) the posterior probability distribution over states (or actions, depending on the mechanism). This is a prerequisite for selecting assistive interventions using decision-theoretic methods (i. e., that aim at maximizing the expected utility).
**F.struct.state**	The state maintained by inference provides a structured representation of the environment state. This allows the formulation of state predicates and the dynamic synthesis of contingency plans. (Otherwise the state typically represents the action currently executed.)
**F.non.monoton**	Non-monotonous action sequences are considered, that – temporarily – may increase goal distance. (This affects the number of plans that need to be considered. Methods using explicit plan enumeration usually avoid non-monotonicity.)
**F.complexity**	Filter step complexity (computational complexity for the filtering step from 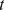 to 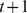 ). If greater than 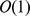 , for instance 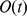 , then online filtering is essentially intractable.
**Method**	Type of inference method used.
**Scenario**	Scenarios considered in experimental tasks.
**N.states**	Number of 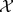 states considered. (See text for further explanation.)
**N.plan.length**	Lengths of plans considered in study.
**N.classes**	Number of classes in classification target used for performance evaluation.
**N.subjects**	Number of subjects participating in trials (or “sim” in case evaluation is based on simulated observations).
**M.accuracy**	Accuracy is provided as performance measure.
**M.conf.based**	Other quantities based on confusion matrices (true–positive rate, precision, etc.) are provided as performance measures.

We note that *N.State* has substantial methodological drawbacks. First, this number (in fact, *any* number quantifying the level of detail of the respective experimental scenario) is often not given explicitly and has to be inferred from the study description. Secondly, if highly discriminative observations are available (such as the ground action label used in some of the simulation studies), only a small portion of the potential state space will have non-vanishing support. In this case, inference in fact only needs to consider a small subset of the state space, based on unrealistic assumptions on observation quality. Finally, a continuous state space obviously would have an infinite number of possible states. Concerning this last point, the real quantity of interest would be the *representation complexity* of the statistical model for the state distribution, as inference algorithms operate on finite representations of distributions. A continuous state space modeled by a Gaussian can be represented by a point 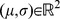 in 2-d space. A categorical state space with 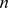 category labels is represented by a point on a 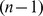-d simplex. So, although the latter state space is finite, its representational complexity is higher than for the continuous Gaussian model. Fortunately, all studies in the literature survey used some kind of categorical state space. Therefore, despite its deficiencies, for the purpose of this study *N.State* was considered as useful surrogate measure for model complexity.

#### 1.3.2 Survey results

21 studies were analyzed in the survey, these include the studies contained in [18]–[20] with the addition of new results we regarded as relevant for the topic of this study. An overview of the analysis results is given in Table 2. There were two studies with more than 100,000 states (studies 6 and 8). 7 studies (33%) considered no more than 1000 states. The median plan length used in trials was 15 (with interquartile range 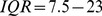). Concerning trial sizes, the median number of subjects was 3 (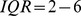).

**Table 2:** Quantitative and qualitative properties of selected studies on activity and intention recognition.

	Reference	F.latent.infty	F.plan.synth	F.duration	F.action.sel	F.probability	F.struct.state	F.non.monoton	F.complexity	Method	Scenario	N.states	N.plan.length	N.classes	N.subjects	M.accuracy	M.conf.based
1	[1] [4]	▪	▪	□	▪	▪	▪	▪	1	B^D^	M	70,000^†^	20	3	23	□	□
2	[2] [56]	▪	▪	□^†^	▪	▪	▪	▪^†^	1	B^D^	OM	–	–	◊	sim	□	□
3	[3]–	▪	▪	▪	▪	▪	▪	▪	1	B^PF^	O	70,000^†^	15^†^	10	6	▪	▪
4	[4] [20]	▪	▪	□	▪	▪	▪	□	*t*	B^Pl^	K	10,000^†^	–	3	sim	□	▪
5	[5] [20]	▪	▪	□	▪	▪	▪	▪	1	B^P^	K	70,000	6	5	sim	▪	▪
6	[57] [19]	▪	□	□	□	▪	▪	□	1	B^D^	A	200,000	5^†^	6	6	□	▪
7	[12] [19]	▪	□	□	▪	▪	▪	□	1	B^D^	K	70,000	40	◊	2	□	□
8	[21] [18]	□	▪	□	□	▪	▪	▪	1	B^D^	O	250,000^†^	–	5	5	▪	□
9	[58] [59]	□	▪	□	□	▪	▪	▪	*t*	N^BN^	M	1,000^†^	–	15^†^	sim	□	▪
10	[60]–	□	▪	▪	□	▪	□	▪	1	B^H^	K	28	6	6	–	▪	▪
11	[61]–	□	▪	□	□	▪	□	▪	1	B^H^	A	300^†^	12^†^	15	3	▪	□
12	[62] [19]	□	▪	▪	□	▪	□	▪	1	B^RP^	K	96	–	13	2	□	▪
13	[63] [19]	□	▪	▪	□	▪	□	▪	1	B^RP^	O	3,500^†^	3	3	2^†^	□	□
14	[64] [56]	□	▪	□	□	▪	▪	▪	*t*	O^ML^	M	–	20^†^	4	14	▪	▪
15	[29] [19]	□	▪	▪	□	▪	▪	▪	1	B^D^	AK	528^†^	–	33	3^†^	▪	▪
16	[65] [19]	□	□	▪	□	▪	□	□	1	N^MH^	O	720^†^	–	2	1	□	▪
17	[66] [19]	□	□	□	□	□	□	□	1	L^DL^	K	–	15	6	sim	□	▪
18	[67] [19]	□	□	□	□	□	□	□	1	L^DL^	AK	–	24^†^	8	3	▪	□
19	[7]–	□	□	□	□	□	□	□	*t* ^2^	O^G^	M	–	50^†^	◊	2^†^	□	□
20	[68] [19]	□	□	□	□	▪	□	□	1	L^P^	A	100^†^	40^†^	7^†^	6	▪	□
21	[8] [18]	□	□	▪	□	▪	□	□	1	B^MF^	A	20,000	14^†^	14	3	▪	▪

“▪”  =  feature included in study.

“□”  =  feature not included.

“*x* ^†^”  =  value/property *x* not explicitly stated in study description.

“–”  =  value unknown.

“◊”  =  property not meaningful considering target of study.

Method codes: *L*: logic-based (*DL*  =  description logic, *P*  =  combined with possibility theory). *B*: using some variant of sequential Bayesian filtering (exact: *H*  =  HMM or extension, *D*  =  other DBN, *Pl*  =  transformation into a planning problem, *P*  =  partially observable Markov decision process; approximate: *PF*  =  particle filter, *RP*  =  Rao-Blackwellized particle filter, *MF*  =  marginal filter). *N*  =  Non-sequential Bayesian inference (*MH*  =  Metropolis-Hastings, *BN*  =  unrolled Bayes Net). *O*  =  other exact method (*G*  =  some kind of grammar, *ML*  =  Markov Logic net). *Scenario* codes: *K*  =  kitchen task, *A*  =  other activities of daily living, *O*  =  office, *M*  =  miscellaneous other scenario.

We consider the first five studies as CSSM-like approaches.

It can indeed be observed that CSSMs have been evaluated only in simpler scenarios, and complex experimental settings have only been used for testing more simple inference methods. There was a single study using a model supporting all features (study 3). Studies including durative actions used at most 70,000 states. As CSSM-like approaches all studies were considered that supported latently infinite state spaces and plan synthesis. From the resulting five studies (studies 1–5), only a single one (again, study 3) used non-simulated observation data and durative actions.

While non-CSSM methods have been evaluated with in median 6.5 (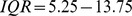) different activity classes, experiments using CSSMs distinguish only 4 (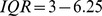) activity classes. When fewer target classes have to be discriminated, it is easier to achieve higher recognition accuracies. The plan length to be recognized by the approaches is also influenced by the supported features, where it can be observed that more complex models are usually evaluated with shorter plans. For instance, approaches employing plan synthesis (studies 1–5, 8–15) have been evaluated with a median plan length of 12 (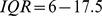), while in median 24 (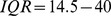) actions had to be recognized without plan synthesis. Similarly when the action's durations are modeled, the plans have a median length of 10 (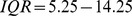), whereas plans of length 20 (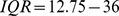) are used when actions are modeled without durations.

The single most frequently used performance measure was accuracy, (10 studies, 48%). 16 studies (76%) used some kind of performance measure derived from the confusion matrix (accuracy, precision, recall, 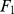, etc.). Four studies reported no performance data. No study used performance measures sensitive to the sequential (or causal) structure of the *sequence* of estimates (cf. Sec. 2.4.2.). The median number of target classes used in performance evaluations was 6 (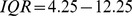). Activities of daily living represented the majority of scenarios (12 studies, 57%). The most frequent single scenario was kitchen activities (8 studies, 38%).

Considering inference methods, if approximate methods were employed and if on-line filtering was possible (i.e., where complexity  =  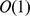), variants of particle filters were used in all cases, with the exception of [8] (study 21). Approximate methods are inevitable if large state spaces have to be supported.

#### 1.3.3 Assessment

While being successful in using CSSMs for intention recognition and state estimation in scenarios of a level of complexity comparable to those reported in Table 2, we made the experience that achieving success with CSSM-based methods in larger settings, such as they occur in certain domains of everyday activities, is not as straightforward as their intuitive appeal implies. When applying the CSSM approach to the scenario outlined in Sec. 2.1.1 (with an average plan length of 91.6 actions), our initial attempt did not yield a model that was able to compete with a simple hidden Markov model applied to the same estimation task. The CSSM models were found to contain several hundred million states. This indicates a potential scalability problem of the method due to combinatorial explosion.

As the above survey results indicate, current studies have considered scenarios of substantially smaller size. For instance, Ramírez and Geffner [5] infer the plan of a (software) agent solving a problem in a kitchen environment (among others). They show how the plan can be inferred when observing the *actions* executed by the agent. The model contains about 70,000 states, including location of four ingredients. There are less than 10 different action classes including interacting with kitchen utensils and the ingredients like frying and mixing. Baker et al. [1] use a Markov Decision Process to model agent or human behaviour. The model describes an agent moving on a 2-dimensional grid towards a goal, avoiding obstacles. The state is the position of the agent and locations of obstacles, resulting in approximately 70,000 states. One of three goals is recognized by Bayesian filtering. Krüger et al. [3] recognize human activities in a presentation scenario. A state in their model contains the location of up to three people and the currently executed activity. They distinguish 10 activities (sitting, presenting, discussing, walking to different locations) using a particle filter. As an example of inference without CSSM modeling, Dai et al. [21] recognize different meeting activities of three to five persons. They manually modelled a Dynamic Bayesian Network with two activity nodes (discussion, presentation, and various sub-activities), and different context nodes (e. g. locations and roles). They use exact inference using an exact Bayes Filter adopted to this particular DBN, showing that 250,000 states can be handled without approximations in this scenario. Accuracies have been evaluated for different nodes independently, distinguishing up to five different activity classes.

While the first two operate only on simulated data and action observations, the last two examples use real sensor data. Nonetheless, none of the scenarios contains fine-grained activities, where more than 10 classes of activities are distinguished. As opposed to 91.6 actions on average for our scenario, the scenarios presented here do not exhibit more than 20 actions that are executed. As a consequence, the results reported in previous studies may not be applicable to the larger scenarios arising from the use of CSSM methods. The question is whether the scalability issues we encountered can be resolved by suitable design decisions. To answer this question, we conducted a study on the feasibility of CSSM methods for reconstructing activity sequences of durative actions from noisy observation data in scenarios with millions of states. Considering the studies discussed above, this is the first attempt to analyze whether the CSSM method is applicable to problems of this scale. We therefore provide important evidence regarding the general applicability of the method. In addition, we provide data on the impact of several important modeling considerations – such as the choice of inference method – on the performance of the resulting system.

### 1.4 Study objectives

Objective of this study was to evaluate the applicability of CSSMs for the purpose of sequential state estimation in dynamic systems with very large state spaces and dense transition models. Focus was the application domain of tracking human activities. Study aims were answering the following two subsequent research questions:

- Is it possible to achieve successful state estimation using CSSM models of everyday activities with large state spaces (containing hundreds of millions of states)?
- Which modeling factors (duration model, action selection heuristics, inference algorithm, etc.) are relevant for achieving a good performance in CSSM-based inference?

These questions were reframed into two research hypotheses:

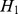
: A suitably parameterized CSSM model for a typical activity of daily living will achieve the same accuracy on average on a given estimation task as a conventional state space model built from training data when applied to the same task.

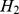
: All CSSM modeling factors and their interactions have significant effects on the average accuracy achieved in state estimation using the CSSM model.

The study focused on the *applicability* of the method, not on *superiority* with respect to other methods considering measures such as accuracy. The primary motivation of using CSSM-based methods is not that it gives a higher performance, but rather that it provides additional benefits for model construction such as use of prior knowledge and reusability (cp. Sec. 1.1). It was also not an objective to build a model with specific reusability properties or to show that CSSMs indeed provide the claimed benefits. A deeper investigation of these aspects is of interest only once it has been established that CSSMs are applicable to the inference task itself.

## Materials and Methods

This section explains the methods and design of the empirical study. The study and all procedures were approved by the institutional review board of Rostock University (A 2014-0057). Participation was voluntary and all participants provided written informed consent. Data presented in this study is anonymous and does not allow identification of individual characteristics of participants.

The roadmap for testing both research hypotheses is: collect real-world data of an activity of daily living, build a suitable CSSM model, and evaluate performance of the model against the recorded data. Sec. 2.1 presents the trial setting, experimental procedure, and annotation methodology. The statistical model used for the experiments is described in Sec. 2.2, including the sensor model, action durations, and the DBN structure. Sec. 2.3 describes the two main inference methods used for the experiments: the particle filter and the marginal filter. The concrete experimental setup (choice of model and parameter combinations) and evaluation methodology for testing the research hypotheses 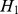 and 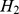 is described in Sec. 2.4.

### 2.1 Empirical data

The use of empirical trial data was chosen for the following considerations:

- Using simulated data (presumably from the same model that is used for inference) will exaggerate accuracy and overestimate the effect of action selection heuristics: if actions are simulated according to the same selection heuristics used for inference, the heuristic essentially *knows* which actions are executed. This is highly undesirable, as it will guide research on heuristics in the wrong direction.
- Evaluating model behavior with respect to sensor data obtainable in real settings requires to have such data available for use as observations.
- As long as we do not know whether prior knowledge provides enough information for building CSSM models, it seems prudent to use samples of real-world behavior as starting points for model construction, in order to arrive at symbolic models that have realistic structural complexity with respect to everyday behavior.
- A certain amount of training data was required for building the baseline classifiers, against which the CSSM model was to be compared.

#### 2.1.1 Trial setting

**Figure 1:**
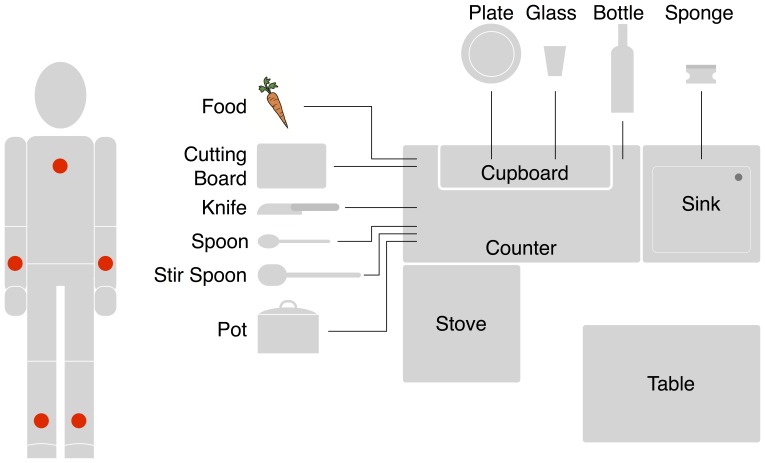
Instrumentation and trial setting. Left: Instrumentation of participants (red points indicate IMU positions). Right: Conceptual spatial layout (view from above) and domain objects of trial setting.

A typical meal time routine was selected as trial setting, consisting of the following major tasks: (i) Prepare meal (prepare ingredients; cook meal). (ii) Set table. (iii) Eat meal. (iv) Clean up and put away utensils. (A symbolic map of the spatial structure of the trial domain and the involved domain objects are given in Fig. 1; a more detailed task sequence is given in Table S1). Selection of this scenario is based on the following considerations:

1. It combines a relevant activity of daily living (eating) and a relevant instrumental activity of daily living (meal preparation and cleanup) [22]. Assisting (instrumental) activities of daily living is an important application domain of assistive systems.
2. It covers as subtask (meal preparation) an activity that is used as functional measure for recording the level of cognitive support required by a person suffering from cognitive decline (the Kitchen Task Assessment, [23]). In addition, there is evidence that action languages can be used to model erroneous behavior specifically for this setting [24].
3. Kitchen activities are frequently used as trial settings for activity recognition methods (see Sec. 1.3.2 and [25]). A successful use of CSSM for this setting should allow many researchers to reproduce the results of this study in a similar environment.
4. The task has a non-trivial causal structure combining regions of high dispersion, where many different routes exist (often caused by permutation effects, when 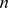 actions can be executed in any order, such as setting the table or cleaning up kitchen utensils) with regions of low dispersion (preparing food and cooking food are strictly sequential), making the use of CSSM techniques meaningful.

#### 2.1.2 Subjects and sample size

Target of the study was the comparison of the CSSM approach to standard methods in a scenario of realistic complexity. Therefore, as merely relative comparisons between methods were required, the representativity of the subjects chosen for the trials was not an issue, allowing the use of a convenience sample of volunteers. As CSSMs are conceptually not built from training data but from prior knowledge, the main purpose of the empirical samples is to provide data for model comparison. Seven subjects were considered sufficient to detect relevant effects on accuracy, such as consistent inferiority of CSSM in comparison to standard methods, at the 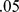 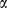 level (significance level). The rationale here is that if CSSM can not be proven to be inferior at this level, then this justifies to spend the effort on a larger scale experiment.

With respect to designing the symbolic component of the CSSM system model, seven data sets were considered sufficient to allow a system designer to detect all relevant causal dependencies. Although there is no direct data on how much experimental data is required for building successful causal models, a weak argument can be found in the domain of usability research, where it is established that in interactive software five to seven subjects are sufficient to identify most usability problems – most situations where system behavior does not meet user expectations [26]. Furthermore, the causal model is not subject to the 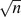 law regarding the standard error of a parameter estimate, as at the symbolic level a single example is sufficient to infer a causal link.

For methodologically obvious reasons, a leave-one-out cross-validation would have been infeasible considering CSSM model construction: it would have required the availability of seven model engineers of identical qualification. Therefore, in order to not place the baseline models at a disadvantage relative to the CSSM model, they also were built on the complete data. Thus, CSSM and baseline performance can be expected to be exaggerated in absolute terms due to overfitting. However, as this study focuses on a comparison of modeling factors, absolute performance is of minor interest. Indeed, as the training-based baseline has more parameters as the CSSM model (cp. Sec. 4.2), this exaggeration should favor the baseline. This bias is actually desirable, as it is an additional safeguard against type I errors considering 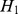.

#### 2.1.3 Experimental procedure

Considering the study objectives, neither absolute motion trajectories nor absolute action duration were of relevance. Therefore, it was possible to use a simplified motion capturing environment where some of the kitchen utensils (for instance, the stove) were replaced by physical props (cp. [27]), and some actions (e.g., cooking) were shortened to bound overall experiment duration. (See Fig. S1 for the physical setup.)

The experimenter presented the experimental task verbally to the participants and explained the stage, the props, and their use. Afterwards, the participants were instrumented with motion capturing equipment. After the “start” signal, the participants would execute the task; the experimenter would monitor task execution and prompt the next step in case the participant got stuck. Within the causal dependencies of actions (food needs to be prepared before being cooked) the participants were free to choose the sequence of actions. The experiment was simultaneously recorded by a documenter on video to enable later annotation.

#### 2.1.4 Sensor data and preprocessing

As prototypic examples for realistic sensor setups, a motion capturing system based on wearable inertial measurement units (IMUs) was chosen. This choice was motivated by the following considerations in favor of other setups such as RFID labeling [28], cameras [18], or various multi-modal setups [29]:

- This sensor setup is used in several experiments by various researchers [30]–[32], simplifying a translation of the CSSM method to other available data sets.
- As IMUs do not require the instrumentation of the environment, they are a technically and economically feasible choice for everyday environments.
- As IMUs monitor a specific individual, identification problems do not arise (although such problems by design did not arise in the trial setting, they become relevant when translating results into the application domain). Likewise, environmental factors such as lighting conditions have no influence.
- If absolute accuracy is not a major target, it is comparatively easy to set up IMU sensor models with reasonable performance.
- Some researchers claim that “wearable sensors are not suitable for monitoring activities that involve complex physical motions and/or multiple interactions with the environment” [19]. It is therefore especially interesting to see whether a more refined system model is able to alleviate these problems.

The participants were instrumented with five IMUs, fixed at lower legs, lower arms, and upper back. These sensor locations were chosen to be compatible with sensor data available from other experiments [32]. For each sensor three axis acceleration and angular rates were recorded, with a sampling rate of 120 Hz. Although provided by the sensing platform, magnetometer readings as well as higher order features (such as joint angles) were discarded, as such features may be not available in low cost equipment. The resulting data stream of 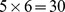 signals was segmented into frames using a simple window-based segmentation with a window size of 128 samples and 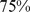 overlap, giving a frame rate of 3.75 Hz. For each frame, mean, variance, skew, kurtosis, peak, and energy were computed for each signal. This stream of 180-dimensional feature vectors at 3.75 Hz was then subjected to dimension reduction by applying principal component analysis to the full set of feature vectors, choosing the loadings of the factors corresponding to the 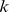 largest eigenvalues as effective observations. (See below for the method of choosing 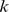.)

Additionally, the collected data was annotated based on the video logs. This is done in order to provide a target label 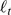 for every observation 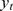 such that methods for supervised learning can be applied. Furthermore, comparing target values with the values estimated from observation data is used for quantifying the performance of the estimation procedure. These labels are called “ground truth”, as they conceptually provide a symbolic representation of the true state of the world at time 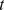. As CSSMs provide causal representation of the human behavior, the underlying annotation has to be causally correct, too. However, in reality, labels are a finite set 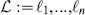 where besides the equality relation no other algebraic structure on 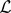 exists. For that reason one has to ensure that the produced annotation represents a causal structure. The detailed procedure of annotating the data and ensuring its causal correctness can be found in Appendix S3. The annotations for subject *S1* are provided in Table S3.

### 2.2 Statistical model

The statistical model formally describes the framework and assumptions of the model for the kitchen task scenario. This section briefly describes the model for the inference LTS (iLTS), including the sensor model, duration model, and action selection heuristics. For a discussion of the difference between the inference LTS and the annotation LTS (aLTS) we refer to Appendix S3. Detailed discussions of the statistical model and its development process is given in Appendix S4. Finally, the baseline models (QDA and HMM) are presented briefly, which are used as comparison against the CSSM model.

#### 2.2.1 CSSM model structure

**Figure 2:**
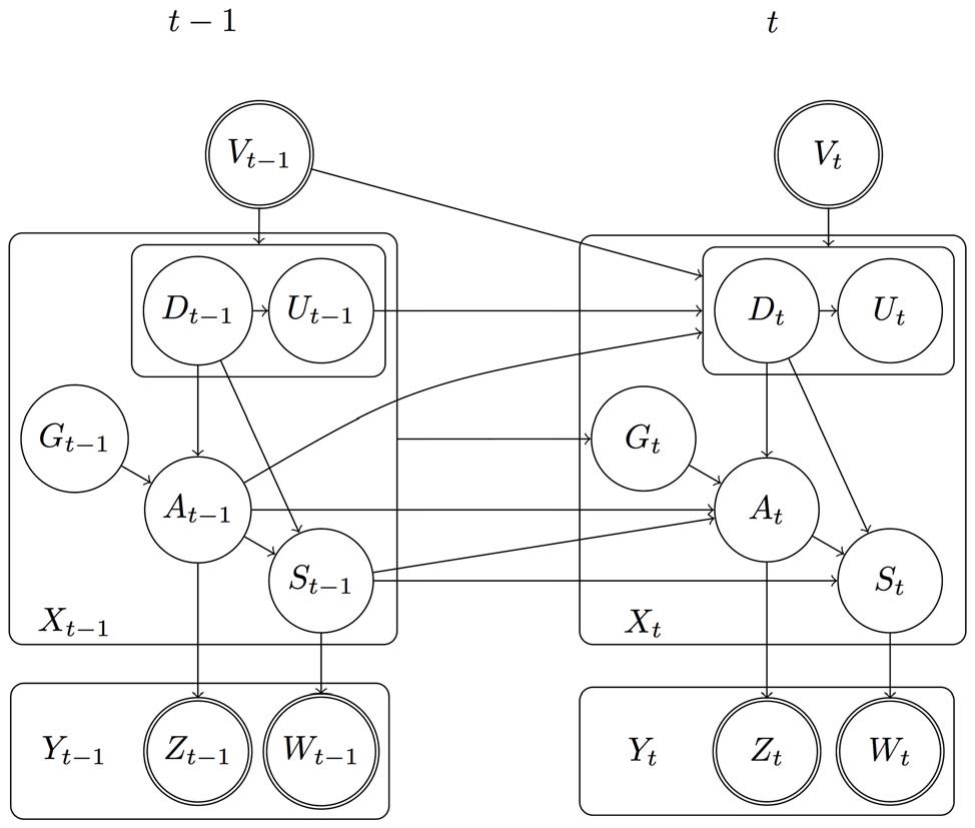
CSSM DBN structure. Boxes represent tuples of random variables. An arc starting/ending at a box ( =  a tuple) represents a set of arcs connected to the tuple's components. Nodes with double outline signify observed random variables.

For the probabilistic model of Sec. 1.2 (see Appendix S2 for details), a DBN with the structure given in Fig. 2 was used. 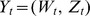 is the observation data for time step 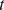, i. e. the sensor data as discussed in Sec. 2.1.4. 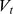 is the associated time stamp, required to be strictly increasing. 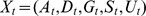 defines the hidden state. For this study, 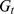, the current goal, could be assumed to be constant, namely that the user has prepared the meal, eaten, and cleaned afterwards. A new action is selected according to the action selection heuristic 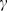, which incorporates the distance from the current state to the goal (for more details on action selection heuristics, see Sec. 4.1.5 of Appendix S4). 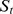 is the LTS state for time step 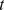: either the result of applying the new action to the previous state, or by carrying over the old state. For the purpose of this study, actions could be assumed to be deterministic and with instantaneous effect. In contrast to the model defined in Sec. 1.2, actions in our model may last longer than a single time step. A model was chosen where multiple observations may correspond to a single action. This model introduces a real-valued random variable 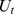 representing the starting time of an action 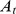 and a boolean random variable 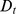 signaling termination status of the previous action 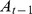.

As the sensor model, all actions 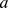 of a given class 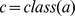 share the same observation distribution, each being a multivariate normal distributions with unconstrained covariance. Although there is no reason to believe that the observation data is particularly well represented by this model, it was found to perform reasonably well in the baseline models, justifying its further use in this study. The 16 action classes and their empirical frequencies can be seen in Fig. S3. For example, an action class is *TAKE*, while the actions belonging to it are *take carrot*, *take bottle*, *take spoon* etc. As alternative to the IMU sensors, a location-based model was set up, giving categorical observations (place names) of the 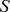 state component. The observations themselves were taken from the annotations of each subject, where the locations of the protagonist (3 places) and the food (6 places) were used (see Table S5).

For simplification it was assumed that all actions of a given class share the same action duration distribution. Note that duration distributions with large, possibly infinite, support increase inference complexity. In order to determine this effect, an instance based and a parametric duration model was built. The instance based model was given by the corresponding empirical distribution function, that is by the observed action durations from the annotations. For the parametric models, the distribution giving the maximum likelihood was selected from a set of candidate distributions (including Cauchy, exponential, and lognormal) the parameters of which were fitted to the observed class durations.

As primary goal-driven action selection feature, the goal distance feature 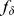 as discussed in Appendix S2 was chosen. As computing goal distances may become intractable for large models, two approximations were considered in this study:

- A goal distance heuristic 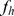, that assigns heuristic distances to LTS states based on prior knowledge, identifying 14 serial task steps there were used to define a map from LTS state 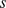 to remaining script steps 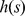.
- A restricted goal distance feature 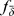. Here, only those LTS states were considered that are visited when using the annotations as exact observations. Restricted goal distance 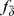 should give an upper limit to the gain achievable by a goal distance measure.

To gain insight on the effect of weight factors, each of these features was tested with the weight values 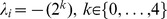, using exponential probing.

#### 2.2.2 Baseline models

Two baseline models for estimating the action class from observation data were built: a quadratic discriminant model (QDA) with one category for each action class and a hidden Markov model (HMM) with one state for each class. The QDA model was constructed from the sensor models 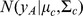 and the priors 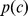, given by the frequencies of the action classes in the data set annotations. The HMM transition matrix was computed by counting the class-transition frequencies in the data set annotations, the 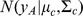 were used as observation model. The baseline models were used to establish target values for estimation accuracy and to select between alternative observation models (see also Sec. 2.4).

### 2.3 Inference methods

Due to the expected size of the state space, exact methods are infeasible and approximate methods for inference were selected. In order to assess the effect of inference method on performance, two methods were compared: a particle filter (PF) and a marginal filter (MF), a variant of the D-condensation algorithm described in [8].

#### 2.3.1 The particle filter

The PF maintains a vector of 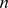 weighted samples 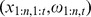 where 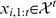 and 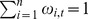 such that the density 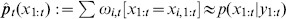 approximates the joint filtering distribution. A standard bootstrap filter was used [33] where the system model serves as proposal function: given a sample vector 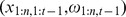 and a new observation 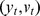, a new sample vector 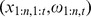 was produced by drawing 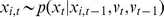, setting 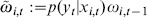 and normalizing 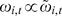. The effective number of samples is computed from the weights as 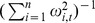 [34]. If this number drops below a threshold, resampling is performed. The filter step complexity of PF is 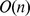. If 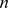 is fixed, this is 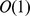.

Essentially, a PF represents the probability of a point in state space by the density of samples in the vicinity of this point. This works very well in continuous state spaces where a meaningful concept of “distance” can be defined. In these domains, all particles typically occupy different points in state space (with the exception of resampling time, when particles are copied); there are as many different state samples as there are particles. In discrete categorical spaces, this does not hold any more. There is no “distance” between points in state. Probabilities have to be represented by particle weights – and as a PF strives for all particles to have equal weight, this results in probabilities to be represented by the number of particles in this state (explaining the “particle clinging” phenomenon often observed in PF applications). In discrete categorical spaces, there are usually much more particles than states. PF thus may perform suboptimal in discrete categorical spaces with high complexity – which are created by CSSM models.

#### 2.3.2 The marginal filter

The MF is tailored towards categorical discrete spaces, where there is no notion of distance, but where there is a chance for trajectories to end up in the same state (consider 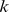 actions that need to be done in any order: there are 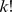 trajectories, all ending in the same state). The MF uses a finite *set* of states for approximating the marginal filtering distribution by maintaining a density 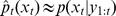 with finite support, 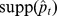. Being finite, 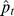 can be represented by a set of ordered pairs (implementationally, these sets were built using tries, see Sec. 4.2.1 of Appendix S4). The value of 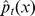 is computed by summing over all trajectories that arrive in 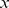 at time 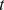. The MF therefore should give a better approximation than the PF in categorical domains, specifically with high dispersion, as here many different trajectories could lead to the same state. MF inference proceeds in two stages. First, prediction 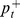 and uncorrected posterior 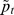 are computed using
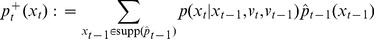
(1)

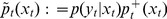
(2)


If 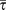 and 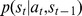 have finite support (specifically, if 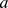 is deterministic), then both computations remain tractable since only a finite number of states is reachable from each state in 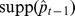. Thus 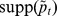 is finite too. The second stage computes 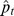 from 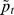. In general, 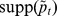 will contain more than 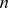 states, requiring pruning. For this study, 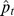 was computed from 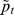 by selecting the 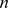 most probable states from 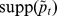, normalizing the values so that 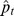 sums to one. The bias introduced by this method was considered negligible for the purpose of this study.

If the number of actions applicable to a state can be considered constant, the filter step complexity of MF is 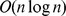, giving 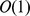 if 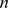 is fixed. The term 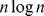 is due to the sorting procedure implicit to the pruning process. For computing the approximate marginal smoothing density 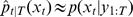 and the MAP (maximum a-posteriori) sequence 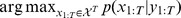 using an adapted Viterbi algorithm [35], a complexity of 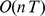 can be achieved.

In general, the support of 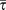 will not be finite. However, the frame rate was considered high enough to render the error introduced by approximating 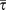 with a suitable point distribution – positioned for instance at the mean or the new time stamp 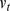 – negligible. For both PF and MF the approximation 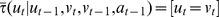 was used in this study. Using the time stamp values 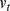 fixed by observation as potential 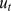 values supports state identification in the MF.

The PF was parameterized with 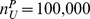 particles. PF resampling threshold was set to 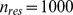 states. The MF used 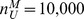 states with discrete empirical timing and 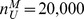 with parametric timing. (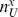 is the number of “representation units” available to filter 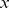.) These MF and PF parameters had shown reasonable results in preliminary tests.

### 2.4 Experimental analysis

The experimental design and evaluation methodology used for establishing the hypotheses 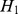 and 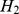 (Sec. 1.4) is described in this section. In this context, an experiment refers to the application of one of the inference algorithms on all data sets, followed by a performance analysis. This includes the set-up of different algorithms (marginal or particle filter) and parameters (e. g. observation models), and describes which analysis methods have been chosen for testing the hypotheses.

#### 2.4.1 Experimental design

The experimental design targeted the following objectives

- Selection of the observation models relevant to analyzing CSSM performance.
- Comparison of CSSM and baseline models (test of 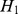).
- Factor analysis of the effect of CSSM configuration parameters on CSSM performance (test of 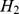).

For model comparison (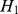), using the best result of some standard parameter search procedure would be sufficient. However, understanding how parameter variations affect model performance and how parameters interact (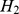) requires a systematic multi-factorial experimental design.

Table 3 lists all factors and levels that determine the experimental configurations resulting from the discussions in the preceding sections. *Target* gives the distribution (or MAP sequence) that is estimated by the inference process. *Model* describes the system model used for representing temporal correlations. *Mode* is the inference mode used for the system model based on the CSSM approach. *Observations* describes the different models for (continuous) IMU observations, using either original or scrambled (ensuring i.i.d. observations, see Sec. 4.1.3 of Appendix S4) sequences, and the categorical location model. For evaluating the effect of 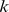, the number of principal components, a selection according to the Fibonacci series was chosen (Fibonacci probing). *Distance*, *Weight*, and *Duration* represent the different tuning parameters of the CSSM system model discussed above.

**Table 3:** Factors and levels for experimental configurations.

Factor	Level	Comment
*Target*	*f*	filtering distribution 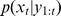
	*s*	smoothing distribution 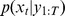
	*v*	MAP-sequence 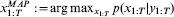
*Model*	*QDA*	(no system model)
	*HMM*	HMM transition matrix
	*C*	CSSM model
*Mode*	*M*	Marginal filter
	*P*	Particle filter
*Observations*	*Oko*	IMU data using 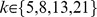 principal components
	*Oks*	IMU data, scrambled
	*OL*	Locations (categorical)
*Distance*	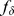	True goal distance, complete state space
	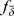	True goal distance, restricted state space
	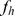	Heuristic goal distance, using script
*Weight*	*L* 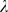	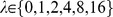
*Duration*	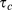	continuous parametric duration models
	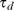	discrete duration models based on empirical distribution function

*Target*, *Model*, and *Mode* are combined to a *Method* factor with the valid levels *QDA*, *HMMf*, *HMMs*, *CMf*, *CMs*, *CMv*, *CPf*, where for instance “*HMMs*” means an HMM model with smoothing distribution as estimation target, “*CPf*” a CSSM model with particle filter and filtering distribution as target, and “*CMv*” a CSSM model with target MAP-sequence computed using the Viterbi algorithm. A detailed summary of all factor meanings is given in Table 3.

Finally, the factor “*Subject*” with levels “*S* 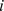
” where 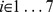 represents the seven data sets being available for experiments.

Using these factors, the following experimental configurations were selected:

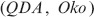
 – Baseline establishing discriminative power of observations. As this model does not use any information on temporal correlations, using original or scrambled observations will have no influence on performance. (4 Configurations)

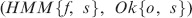
 – Baseline for establishing the impact (i) of information on temporal correlations, (ii) of different observation models, and (iii) of scrambling. The outcome of these experiments was used for selecting the relevant observation models for the CSSM experiments. For each target, the best performing HMM baseline was selected as basis of comparison for the CSSM experiments. (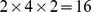 Configurations)

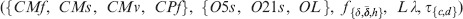
 – CSSM evaluation experiments. The restriction to 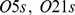 results from the baseline analysis. *OL* implements an observation model based on locations of two objects. For comparing marginal filter and particle filter, it was considered that already the *f* target would allow a substantiated judgment. This results in 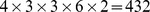 configurations.

A within-subjects design was used where each configuration was applied to all of the data sets, resulting in 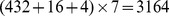 experiments. (We report these numbers to provide an intuition for the number of data points shown in the plots of Sec. 2.4.2.)

#### 2.4.2 Evaluation methods

Performance comparisons for testing research hypothesis 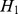 were based on point estimates for the action class at time 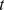, given by the obvious method. (This means choosing the most probable action class for each time 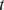 given the estimated filtering resp. smoothing distribution for *f* and *s* targets. For the MAP sequence the action class at time 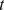 is directly given by the action selected for time 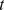.) The estimates were collected into a confusion matrix 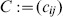, where 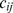 is the number of time-steps where the action class was actually 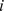 and action class 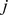 has been estimated. From the resulting confusion matrices, the accuracy 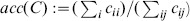) was used as primary comparison measure. The target “action class” was chosen in favor of the more complex targets such as “ground action” in order to allow the training-based baseline models (HMM) a sufficient amount of training data for estimating the transition model. For the QDA model, a finer grained target than provided by the observation model would have been meaningless anyway as the QDA model does not support temporal information and therefore can not disambiguate actions indistinguishable by the observation model.

Concerning research hypothesis 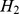, multi-factorial repeated measures analysis of variance (rANOVA) was used to analyze the effects of the configuration factors *Mode*, *Observations*, *Distance*, *Weight*, and *Duration* on *Accuracy* for CSSM configurations.

Accuracy corresponds to assuming a 0-1-loss function for a decision theoretic comparison of classifier performance, where the loss of a classification decision at time 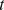 is counted independently of previous and following decisions. If no additional knowledge is available, this correctly reflects system performance. Accuracy is also the performance measure most frequently used in present research (cp. Sec. 1.3.2). Therefore, it was selected as primary measure for comparing model performance.

Although it has the advantage of being a well established and widely understood performance measure, Accuracy has the drawback that it assigns the same value to causally different sequences. Consider the true sequence (on, off, off) for which two hypothetical estimates 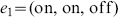 and 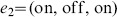 are given. Both estimates have the same accuracy (2/3), but their causal consequences are different. In addition, 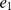 consists of two actions, while 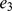 has three actions. Thus, 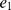 can be regarded as “better” than 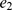, since 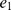 mistakes just the action durations while 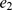 gets the causal sequence wrong. Metrics for measuring sequence alignment – such as edit distances (e.g., the Levenshtein distance) or dynamic time warping (DTW) – may provide a more appropriate measure with respect to these considerations (cp. [36]). However, as it is not yet known in how far the implicit assumptions of these metrics agree with the reality of the application domain considered in this study, performance comparisons focused on accuracy. (A short analysis of edit distance and DTW is given in Fig. S7.)

Besides estimating the action (class) sequence, the CSSM method also allows to estimate the probability of states and, more generally, the truth value of predicates on state. To provide data on the validity of the estimates obtainable, three sample predicates corresponding to three situations of potential interest were established:

- “Danger” – a potentially dangerous situation exist (i. e., the stove is on): 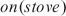.
- “Eaten” – the protagonist has finished the dinner: 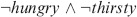.
- “Success” – the protagonist has finished the complete routine (and may be engaged with additional cleanup): 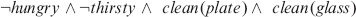.

The ground truth for these predicates was computed by the same method used for generating the location observations. This ground truth 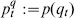 is a Bernoulli distribution with parameter 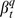 giving the probability of 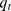 being true. Usually 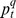 is deterministic with 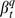 either 0 or 1, except for situations where the aLTS model is ambiguous given the observed actions.

The estimated filtering probability 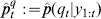 of predicate 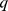 being true at time 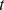 is given by the parameter estimate 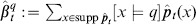. For the smoothing target, 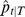 is used in place of 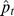. For comparing the estimated distribution 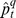 with the true distribution 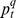 the Jensen-Shannon distance 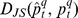 was used. (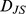 is the square root of the Jensen-Shannon divergence [37].) As measure for the *sequence* of distributions 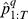 the mean Jensen-Shannon distance was used, given by 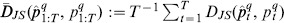. In addition, a conventional accuracy value was computed by 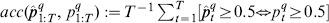. This uses point estimates, assuming 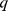 to be true if its probability is at least 0.5.

## Results

This section provides results of the experiments as described in Sec. 2.4. First, some empirical data on the model and the annotations themselves are given. They provide some intuition about the complexity of the experimental setting. Then, the performance of the baseline models is compared with the CSSM models and inference algorithms. The key result is the confirmation of hypothesis 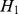. Hypothesis 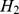 can be accepted after assessing the influence of the parameters (factors presented in Sec. 2.4.1). Finally, results on the ability of state predicate estimation are given.

### 3.1 Empirical data

#### 3.1.1 Data sets and annotations

The data sets obtained have a mean length of 91.6 (standard deviation 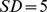) action steps and a mean duration of 950 (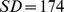) observation steps (cf. Table S2). The shortest observed plan had a length of 66 causal actions (that is, not counting “*WAIT*” actions). This is considerably longer than has been found in previous research (cp. Sec. 1.3).

Plots of the preprocessed observation data (PCA transformed) and action classes for the ground truth annotation are given in Fig. S4. The detailed annotations for subject 1 are given in Table S3.

#### 3.1.2 aLTS and iLTS model

The aLTS model defines 16 action classes and 82 action instances. Thus performance evaluations would be based on 16 target classes. This is in the highest quartile of *N.classes* and it exceeds the number of target classes that have been considered in previous studies on CSSMs (cp. Sec. 1.3.2). The action classes occupied between 0.5% of total time (*TURN_ON*, *TURN_OFF*) and 19.8% (*WASH*). See Fig. S3 for details.

In the iLTS model, these were translated into 44 action schemata (of which 9 were parameterized), resulting in 99 ground actions, produced by the instantiation of action schemata with iLTS domain constants (objects and slot values). The iLTS model defines 18 state variables based on 14 domain objects and 11 slot types (not all objects use all slot types). For further details, see Table S4. In total 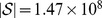 iLTS states were reachable from the initial state using simple breadth-first search (bfs). The median branching factor was 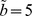 (interquartile range 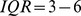). Bfs tree depth was 66 steps, which means that all action sequences taking 66 or more steps will allow all iLTS states to become possible. (The fact that the shortest observed action sequence took 66 steps is pure coincidence.) If all actions have a non-zero probability for a single-step duration (which is the case for parametric duration models), this means that already after 66 *observation steps*, all states have non-zero probability. The maximum goal distance was 40.

The observed sequences traversed 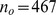 iLTS states. The maximum goal distance in the LTS restricted to these 467 states was 52. For both full and restricted iLTS state space, the maximum goal distance was considerably smaller than the shortest observed action sequence (66). The maximum possible number of iLTS states would have been 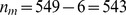 (the plans of all subjects share only the starting state and the final state), the minimum would have been 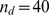 (the length of the shortest path to the goal from the starting state). It is possible to compute a “proportion of unique state discoveries”, given by 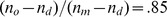. Finding 85% unique iLTS states in the observed data can be taken as indicator of a substantial degree of freedom in task execution provided by the experimental setting.

#### 3.1.3 Action durations

For the duration modeling as described in Sec. 2.2.1 and Appendix S4, the lognormal model provided the best fit for the pooled action durations (although this fit is far from perfect, see Table S6), justifying its use as baseline for determining the set of classes with specific duration models. Ten classes were found, four using Weibull models, two gamma, and four lognormal. The other six action classes shared a common lognormal model. (See Table S7 for additional detail.)

Considering the instance based timing models, a median number of 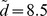 (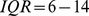) different durations for actions was found. Combining the median number of durations with the median branching factor and the number of iLTS states gives as approximation for total number of states governing inference complexity 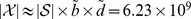.

### 3.2 Model comparison results

Below we provide results for the baseline models (QDA and HMM) as well as for the CSSM model. The performance is always given as accuracy as described in Sec. 2.4.2. For notational conventions, please refer to Appendix S1.

#### 3.2.1 Baseline performance

**Figure 3:**
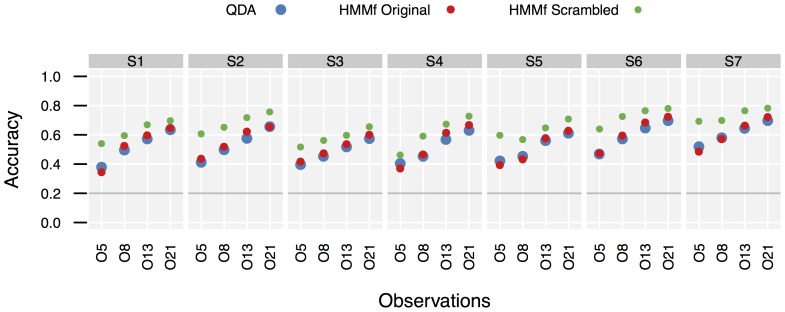
Baseline classifier accuracies per subject. Different numbers of principal components in the observation model have been used. An accuracy of .2 (solid gray line) is achieved by selecting the action class with highest prior probability.

Best mean performance of QDA was 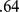 with confidence interval (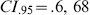). For HMM forward filtering, best mean performance was 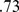 (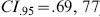) using scrambled observations, and 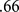 (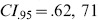) with original observations. In all cases, the number of principal components 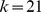. Fig. 3 contains detailed data for all configurations and all subjects. For QDA, scrambled and original observations gave identical performance (as they should). Following, “pp” refers to “percentage points”. An accuracy increase from .27 to .32 is an increase by 5pp.

For HMM, scrambled observations uniformly outperformed original observations for all 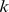 (median increase 9.89pp, Wilcoxon signed rank test, 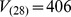, 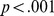). A median improvement of 11.7pp over QDA was found for HMM with scrambled observations (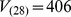, 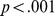), while an HMM with original observations achieved only an improvement of 1.67pp over QDA (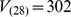, 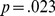).

The observed effect of scrambling was supported by the finding that for the original data there was a highly significant influence of observation position in a run on expected probability, substantiating the conjecture in Sec. 4.1.3 of Appendix S4. This influence could not be found in scrambled data. Consequently, CSSM analysis focused on scrambled data. (See Fig. S2 for additional details).

With respect to 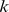, the models using 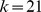 uniformly performed best, while 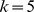 had the lowest mean and median performance. The CSSM analysis was restricted to these two extremes. For completeness we note that *HMMs* (smoothing) outperformed *HMMf* (filtering) with a mean increase of 3.98pp (paired 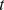-test, 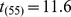, 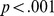), on scrambled data alone an increase of 5.57pp was found (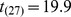, 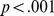).

#### 3.2.2 Comparison between CSSM and baseline: 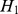.

**Figure 4:**
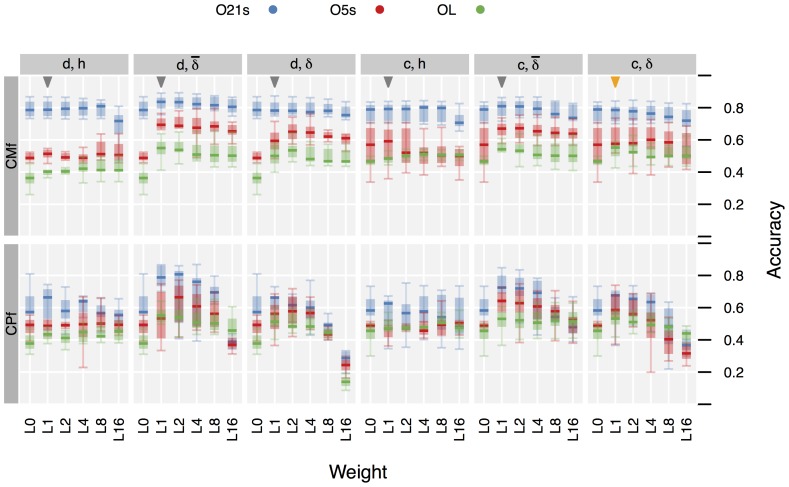
Boxplots of Accuracy vs. CSSM configuration. 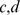
  =  continuous (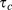) resp. discrete (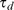) duration model. 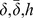  =  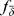 (restricted), 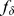 (complete), 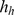 (scripted) distance computation. Details for the *O21s* configurations marked by triangles are shown in Fig. 5. The orange triangle marks the *CMf* configuration used in testing 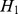.

**Figure 5:**
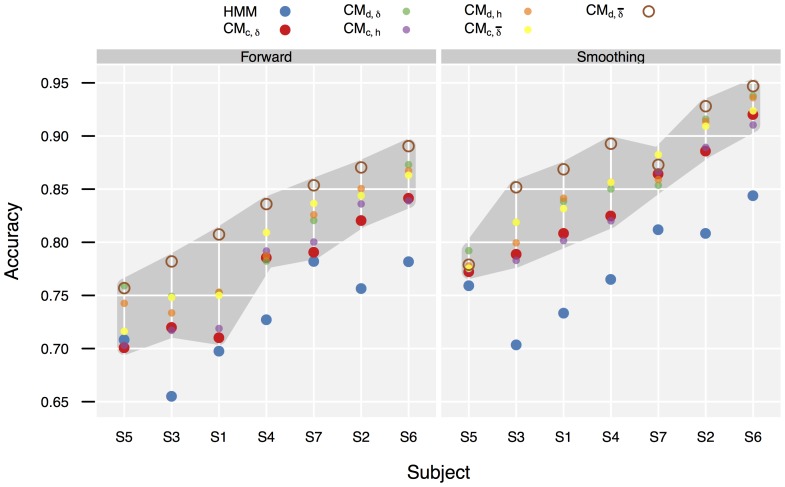
Accuracy comparison of selected CSSM configurations to baseline (HMM), by subject and filter method. c/d  =  use of 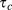 (continuous) or 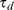 (discrete) duration model. 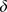/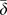/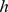  =  use of 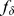 (complete), 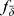 (restricted), or 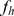 (script) goal distance model. Observation model *O21s*, distance weight L1. (Subjects sorted by median performance in all configuration.)

Fig. 4 gives the performance data resulting from the *CPf* and *CMf* CSSM model configurations. (The complete data, including the *CMs* and *CMv* configurations, is given in Fig. S8). For baseline comparison, the (*CMf*, *O21s*, *L1*) and (*CMs*, *O21s*, *L1*) configurations were selected. The detailed performance data obtained for these configurations is given in Fig. 5. An analysis of the clearly visible influence of the configuration parameters on performance is given in Sec. 3.3.

**Figure 6:**
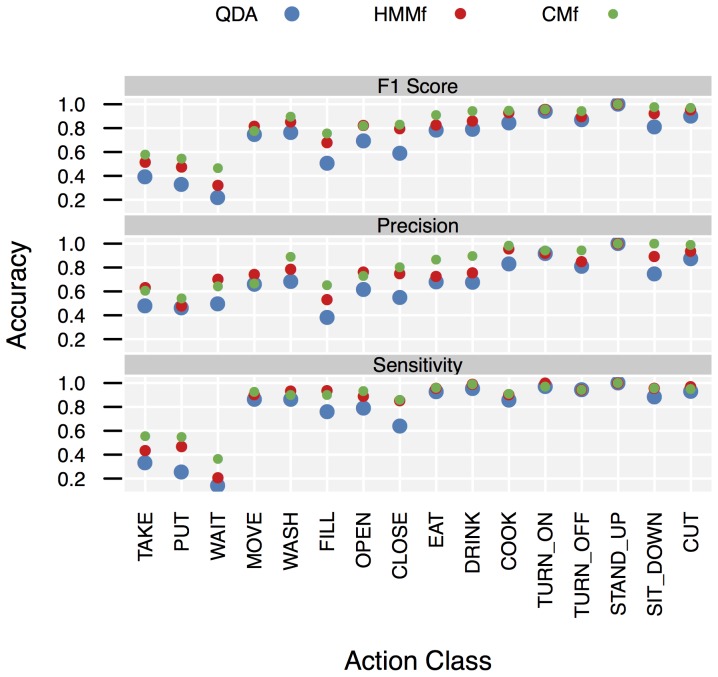
Per-class performance measures. Detailed accuracies for the configuration (*OS21s*, 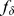, *L1*, 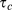).

For testing 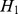, the configurations (*CM* {*f*, *s*}, *O21s*, 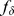, *L1*, 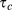) were chosen. These configurations use the least amount of information from the training data, are the least restrictive considering the state space, and employ the most rational action selection heuristic available in this study. Comparison of *HMMf* and *CMf* showed a significant median increase of accuracy for *CMf* by 3.63pp (Wilcoxon signed rank test, 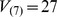, 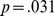). For *HMMs* and *CMs* a median increase of 6.78pp was found (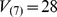, 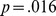). Both results do not support the hypothesis that CSSM models perform at the same level of accuracy as training-based models, instead they suggest that CSSM performance exceeds baseline performance. (For comparisons of the other configurations, see Table S8.) This is also supported by the per-class performance data shown in Fig. 6. (The confusion matrices from which this data has been computed are included in Fig. S5.) Considering the per-class performance there was no strong difference in overall classification behavior between QDA, HMM, and CSSM. However, the 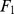 score suggests an overall more balanced performance for CSSM. We also see that there are fundamentally “difficult” action classes (*TAKE*, *PUT*, *WAIT*). Plots of the estimated action class sequences versus ground truth for all (*O21s*, 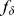, *L1*, 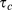) configurations and the corresponding baseline results are given in Fig. S6.

Additional evidence for the alternative to 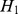 is given by computing Cohen's 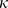 from the confusion matrices and testing for significant difference of the values obtained. The results show highly significant difference in 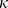 (cf. Table 4). However, as these results are based on using 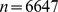, the observed frames, which are typically *not* i.i.d. (independent and identically distributed), we think they exaggerate the true performance difference.

**Table 4:** Cohen's 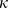 and overall accuracies for selected configurations.

	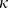	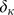	Acc.	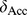
QDA	.6		.65	
HMMf	.7		.73	
CMf	.74	.042	.77	.037
HMMs	.75		.78	
CMs	.82	.072	.84	.064

Cohen's 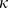 and overall accuracies for selected configurations. 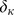, 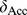  =  difference between 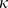 and Acc values, respectively, for CSSM and corresponding HMM (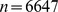, 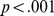 in both cases). Configuration (*O21s*, 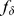, *L1*, 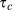).

### 3.3 CSSM configuration factor analysis

The subsequent sections analyze the effects of the different parameters of the CSSM model on accuracy. After describing general observations on parameter interaction and influence, results for the inference methods (*Mode*, particle and marginal filter) and goal distance heuristics (*Distance*) are presented in more detail.

#### 3.3.1 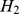: Configuration factor effects

Fig. 4 suggests that several effects of CSSM configuration factors on *Accuracy* are present. These effects were analyzed using rANOVA. The complete rANOVA results including 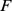 statistics are given in Table S9. Here we concentrate on significant effects with at least medium effect size (using the criterion 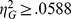; 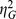 is the generalized Eta-squared effect size measure [38], the medium effect size threshold 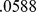 has been taken from [39]).

**Figure 7:**
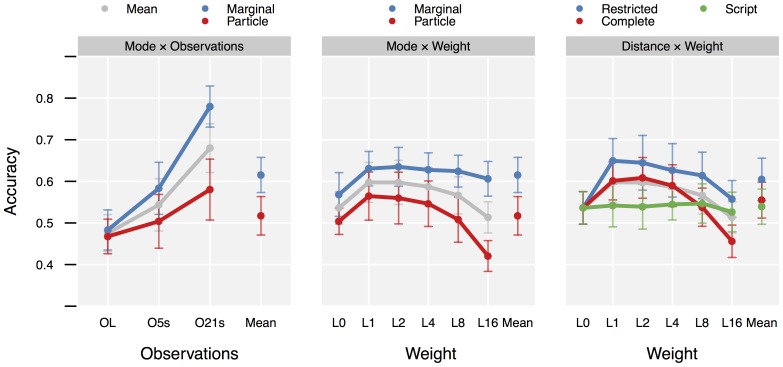
Interaction plots for 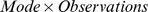, 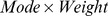 and 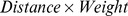. Color: first factor, X-axis: second factor. X-position “Mean”: main effects of first factor. Grey points: main effects of second factor. Error bars give the 95% confidence intervals due to *between subject* variance. Effect comparisons are based on *within subject* differences. Confidence intervals for effects therefore are much smaller than implied by these error bars.

Significant main effects (all 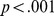) were found for *Mode* (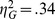), *Observations* (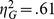), *Distance* (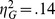), and *Weight* (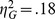). There was no significant effect for *Duration*. Furthermore, there were significant interactions (all 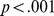) for 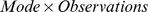 (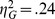), 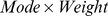 (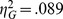), 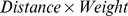 (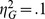), and 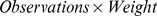 (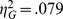). Fig. 7 shows interaction plots for the first three effects. Fig. 4 illustrates the effect of the interaction between 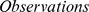 and 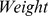 in more detail.

Use of *Marginal* mode increased *Accuracy* over *Particle* mode by 9.81pp (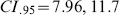). The difference between *O21s* (highest) and *OL* (lowest) was 20.5pp (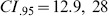). Using *Restricted* distance instead of *Complete* improved performance by 4.99pp (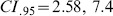), while using *Script* caused a decrease of 1.57pp (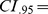
−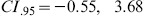). A moderate nonzero *Weight* improved performance, the difference between *L0* and *L2* was 8.41pp (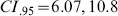). The small difference between *L1* and *L2* was not significant (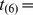
−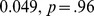). Interestingly, as weights get larger performance begins to decrease again: there was a significant drop from *L2* to *L16* by 8.39pp (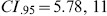).

Considering interactions, the *Marginal* mode gained more from better observations than the *Particle* mode. While there was only small performance difference at *OL* of 1.55pp (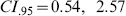) (although significant (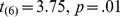)), the *Marginal* mode clearly exceeded the *Particle* mode at *O21s* by 20pp (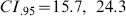). With regard to *Distance* there was a significant effect for transiting from *L0* to *L1* for both *Complete* and *Restricted* with an increase of 6.5pp (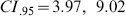) and 11.3pp (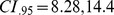), respectively. The tendency for *Script* was not significant (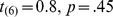). There was a marked decrease in performance of 15.2pp (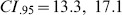) for *Complete* when proceeding from *L2* to *L16*. The same tendency could also be observed for *Restricted*, with a decrease of 8.69pp (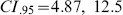). An analysis of the significant interaction of 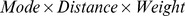 (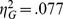), Fig. S10, shows that this tendency is mainly caused by 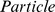 in combination with 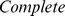 (decrease of 26.7pp (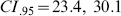) for *L16*) and 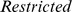 (decrease of 15.4pp (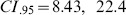) for *L16*). For 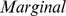 this effect is only significant for *L16* (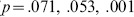 for *L4*, *L8*, and *L16*) for 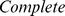 and not significant for 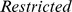 (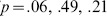).

Concerning the effect of *Script*, a detailed look at the significant interaction between *Observations*, *Distance*, and *Weight* (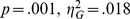), Fig. S9, shows the situation to be more complex. While for *O21s Script* had only significant effect for *L16* (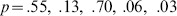 for all five non-zero weights), it had a significant benefit for *OL*, giving an increase of 3.04pp (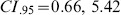) at *L1*, up to 5.58pp (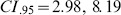) at *L16* (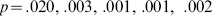 for the non-zero weights). It could thus be observed that with weaker observation models even less perfect distance models begin to show a positive effect.

#### 3.3.2 Understanding the effect of *Mode*

One explanation for the superiority of the *Marginal* mode is that the marginal filter is able to maintain more states than the particle filter, as it represents state probabilities by weights rather than replication counts (cp. Sec. 2.3). To analyze this, the number of LTS states (elements of 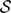) as well as the number of inference states (elements of 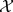) were counted for each step in each filter run. The numbers obtained were compared with the number of representation units (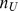) available to the respective filter, giving the quantity “LTS state per representation unit” (SpU) and “inference state per representation unit” (XpU).

Table 5 gives the median values across all runs. The marginal filter clearly makes much better use of the available representation resources. The numbers show the marginal filter to be 80 to 125 times more efficient than the particle filter considering representation unit use. Concerning XpU, the ratio is always 1∶1 for the marginal filter (detailed data is provided in Fig. S11). The row “XpS” gives the number of inference states per LTS state. The marginal filter is able to maintain more inference states (more variations in starting times and action under execution) per LTS state. #S and #X give the median values for the absolute numbers of states. (Note that the particle filter has been used with 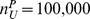, while the marginal filter used 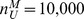 and 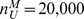.)

**Table 5:** Median SpU and XpS values and ratios, across all runs.

	CMf	CPf	ratio (CMf/CPf)
SpU	0.10	0.001	83.18
XpU	1.00	0.008	127.15
XpS	9.77	6.947	1.42
#S	15000.00	786.500	18.42
#X	1090.50	106.750	10.43

#### 3.3.3 Understanding the effect of *Distance*

**Figure 8:**
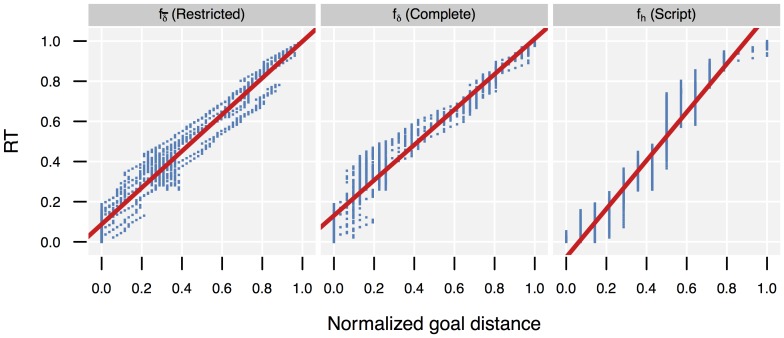
Observed normalized remaining time to goal (RT) versus normalized goal distances. Data based on 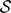 states traversed in observed sequences. These states have been entered at different times by different subjects; due to alternative paths also multiple discoveries by the same subject were possible. In total, 960 different discovery events were recorded (blue points in plot). For each discovery event, we plot normalized goal distance of discovered state (X axis) versus remaining execution time (Y axis). The three plots show the values for different goal distance computation methods/heuristics. The red line is the linear model predicting (*RT*) from heuristics using 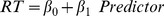. Table 6 gives properties of linear models.

For a state 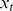 entered at some time 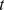 in an execution sequence of total length 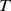, the remaining execution time is given by 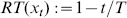. To understand the effect of *Distance* on *Accuracy*, linear models were built that predicted the 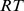 values observed for the ground truth state sequences from the normalized goal distance value given by the different *Distance* methods. The ground truth state sequences were computed using the method described in Sec. 4.1.3 of Appendix S4. Fig. 8 gives the resulting models. All models explained a substantial amount of 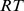 variance. The goal distance values computed by the tested methods thus were highly correlated with the true temporal structure of the observation sequence, although the ground truth state sequences did not follow the shortest paths.

However, while 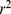 for all methods was high, they showed a markedly different performance (cp. Sec. 3.3.1). As 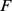 tests comparing the residual variances show (column 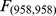 in Table 6), the *Complete* and *Script* models had a significantly higher residual variance than the *Restricted* method in predicting 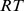. The difference between *Script* and *Complete* also was significant (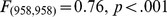). The residual variance of the methods seems to be an indicator for the observed effect of distance method on performance.

**Table 6:** Properties of linear models in Fig. 8 (for all 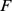, 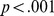).

Predictor	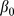	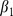	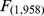	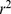	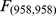
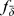 (Restricted)	0.09	0.91	17579	.95	1.00
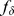 (Complete)	0.13	0.88	13441	.93	0.78
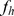 (Script)	−0.08	1.20	10029	.91	0.59

### 3.4 State predicate estimation

**Figure 9:**
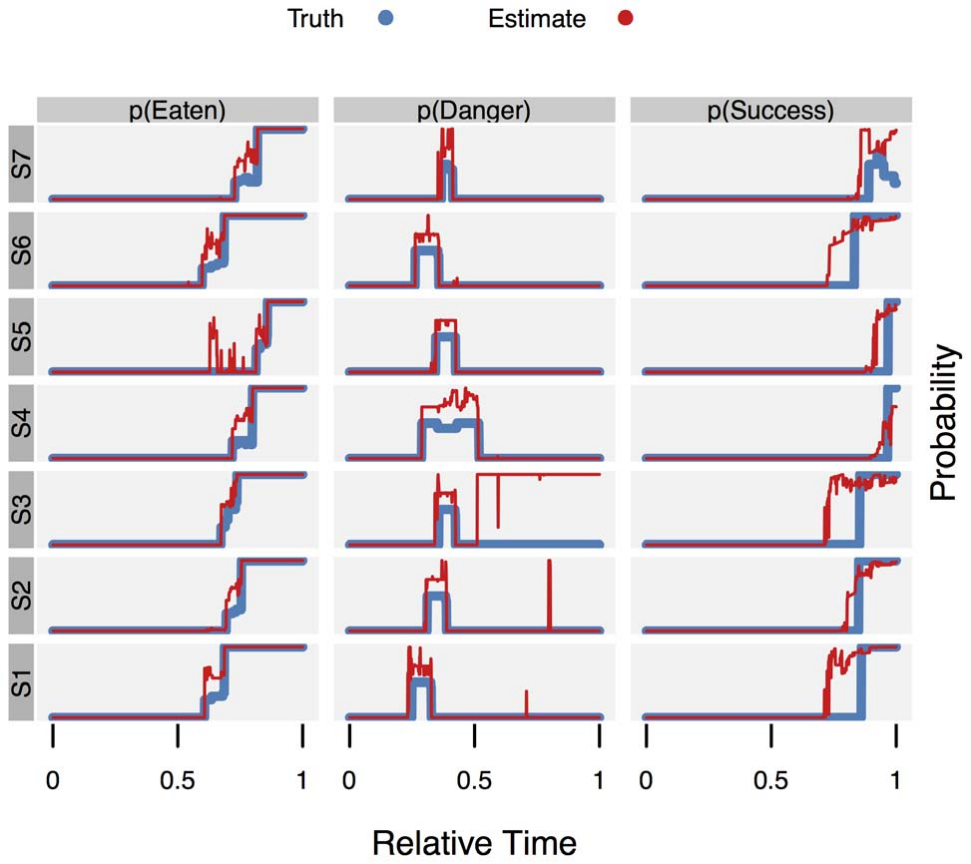
Estimating the probability of state properties. Sample values for configuration 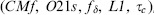, plotted for each subject individually.

Table 7 gives the median Jensen-Shannon distances (JSD) and the accuracies obtained for estimating the probabilities of the selected state predicates “Eaten”, “Danger”, and “Success”. A sample of estimates for the configuration 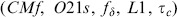 is shown in Fig. 9. In general, predicate estimation achieved median accuracies of .93 or better, reaching perfect estimation for 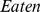 in the configuration 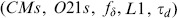 (Table 7). As suggested by Table 7, a significant correlation between JSD and *Accuracy* was observed (Spearman's 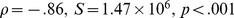). (This correlation is even more prominent in the detail data provided in Fig. S12.) This suggests that accuracy can serve as a surrogate measure, although JSD, being the more sensitive measure, was found to detect differences in situations where accuracy signaled perfect estimates.

**Table 7:** Median values and IQR (Q1, Q3) for Accuracy and JSD found for predicate estimation based on configurations 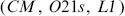.

Predicate	Target	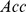	Q1	Q3	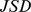	Q1	Q3
Eaten	Forward	.93	.92	.95	.021	.019	.026
Danger	Forward	.99	.98	.99	.017	.015	.023
Success	Forward	.95	.93	.98	.043	.028	.056
Eaten	Smoothing	1.00	1.00	1.00	0	0	.001
Danger	Smoothing	1.00	.96	1.00	.002	0	.038
Success	Smoothing	.97	.89	.99	.033	.017	.064

There was an interesting outlier concerning the estimation of predicate “Danger” for Subject *S3*. Fig. 9 shows a situation where sensor data has been mistaken for *TURN_ON*, causing inference to consistently give the wrong situation estimate. (In an assistive setting, a system would negotiate the true state with the protagonist in such a situation.) This is an instance of a situation where an error in estimating the action sequence with a low impact on accuracy may have a high (non-linear) impact on derived estimates that are based on the causality of the estimated sequence (cf. Sec. 2.4.2 and Fig. S7).

## Discussion

We have introduced CSSMs as an emerging knowledge-based method enabling probabilistic inference in dynamic systems with large state spaces by using computational action languages. As discussed in Sec. 1.1, research on activity and intention recognition considers CSSMs as they potentially allow to build detailed and reusable system models for probabilistic inference. We have claimed that, however, until now there is no empirical proof that CSSMs are indeed applicable to this problem, as empirical studies so far have focused scenarios with limited complexity. Our objective therefore was to move a step further towards providing this empirical proof and to gain insight into the impact of specific modeling aspects on resulting performance. Following, we discuss what our results imply with respect to this objective.

### 4.1 Problem size

While CSSM methods are considered to have interesting benefits (cf. Sec. 1.1), they are liable to produce large state spaces. Our results indicate that modeling activities of daily living, at the level of individual actions using CSSM methods, results in state spaces substantially larger than previously considered in CSSM research (and, indeed, in research on activity recognition and intention recognition in general). Already the state space produced by the domain objects and their possible locations (Table S5) exceeds the largest state space found in previous studies (cf. Table 2) – although this set of objects and locations contains no unnecessary complexity. Overall, we arrived at 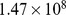 LTS states, corresponding to 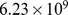 states in 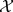 (Sec. 3.1.2). This state space is more than 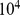 times larger than considered in previous empirical studies on activity or intention recognition.

Considering the observed action sequence length, a median plan length of 15 actions used in previous studies is substantially smaller than what we found for our domain (at least 66 causal actions, mean length 91.6 actions). Given a domain with a specific branching factor, if plans are too short they will access only parts of the state space and therefore also mask inference complexity.

We therefore think it is justified to say that in previous studies the true inferential challenge arising from the use of CSSM methods – maintaining tractable inference in large state spaces – has been masked by the use of simplified experimental scenarios.

Inference with billions of states needs approximative solutions. It is important that the approximation strategies make their errors where they do not matter. The smaller the state space used in the experiments is, the less visible the effect of suboptimal approximation strategies will be. Using small state spaces therefore is dangerous, as it will hide potential flaws of the methodology used (or its application). This is consistent with the observation that approximative filtering in intention recognition and activity recognition usually relies on particle filtering (cf. Sec. 1.3.2), but usually no issues with this are reported – in disagreement with our findings. Specifically studies on CSSMs need to acknowledge this, as here state spaces used in studies so far tend to be even more restricted.

### 4.2 Applicability of CSSMs

Using computational action languages, it is easy to model systems that have very large – even latently infinite – state spaces. The question here is whether such models still afford a meaningful inference. A reasonable requirement is that in case a set of categorical classification targets is given (e.g., the set of action classes), a CSSM should, with respect to these targets, perform at the same level as a simpler system that uses only these targets as state space. If this is the case, using the larger CSSM state space does not – at least not obviously – incur any performance penalty regarding “simpler” estimation tasks, while still providing the benefits of CSSMs.

This has been tested by comparing the accuracy of a CSSM-based method in estimating the sequence of action classes to the performance of HMM built purely from training data. Our findings suggest that a CSSM is able to provide the same level of performance as an HMM on this task, even though the HMM has been allowed to overfit to the training data (the HMM has been built from the complete data set). Indeed, our results in testing 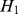 indicate that the CSSM outperforms the training based model (Sec. 3.2.2).

In conjunction with the discussion above, this suggests that the first research question of this study can be affirmed: CSSM models of real world activities afford tractable inference based on data from noisy and ambiguous sensors. This means, it is possible to use symbolic knowledge on the causal structure of human actions to build probabilistic filters which are, considering the computational complexity of the marginal filter, able to work in real time. Notably, this seems to be achievable without posing contingent constraints on CSSM model complexity. This suggests that there is a certain latitude concerning the number (and value domain cardinalities) of state variables built into a CSSM model. As increasing state space complexity does not visibly reduce performance, creating a rich state space by the anticipatory inclusion of state variables of potential relevance in future applications (or different deployment settings) becomes a viable option.

An HMM might not seem to be a very sophisticated adversary. However, for a 16-state HMM, as has been used for this study, the 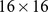 transition matrix is defined by 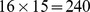 parameters. In contrast to this, the only parameters of the CSSM transition model are contained in the duration model, which in this case amounts to 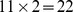 parameters. Therefore, interestingly, the HMM indeed had more degrees of freedom to adapt to the data than the CSSM. (Nevertheless, we think there are other data-driven models, such as Echo State Networks [40], which will provide a much bigger challenge to the CSSM method with respect to sheer performance.)

With respect to the discussion of different performance metrics, which we have only touched in passing (Sec. 2.4.2 and Sec. 16), we fully agree with [36]: for a thorough assessment of sequential classifier performance, its is important to consider criteria such as sequence alignment in addition to measures based on confusion matrix analysis. Otherwise, important aspects – such as the causal structure of the estimated sequence – will be hidden in the evaluation.

### 4.3 Impact of modeling parameters

Considering design decisions, there were two major factors determining model performance:

- Making sure that observations are i.i.d. (independent and identically distributed). This has been the major insight from the baseline analysis. Without i.i.d. observations, a model including temporal structure shows essentially the same performance as a simple QDA. Making observations i.i.d. by using a simple scrambling mechanism improved performance by 9.9pp.
- Using the marginal filter (Sec. 2.3.2) that represents state probabilities by state weights rather than by state duplications (as the particle filter) increases performance by 9.8pp.

We think a combination of these two factors has been the reason for the failure of our initial attempt to build a working CSSM inference system. In hindsight, it is obvious that both aspects have an impact. However, it had not been anticipated how *large* this impact is in comparison to other modeling decisions. Indeed, the analysis in Sec. 3.3.2 shows that the particle filter is not very well suited for state estimation in CSSMs. As outlined in Sec. 2.3 we think the reason is that CSSM state spaces are essentially non-metric. It is interesting that, while present research on activity and intention recognition unanimously uses particle filters if approximate methods are applied (cp. Sec. 1.3.2), only in one case (study 21, [8]) a potential mismatch between this approximation method and the application domain of categorical state spaces is indicated. Our results strongly confirm these findings.

In this context, it was interesting to see that replacing continuous by discrete timing did not have any major impact on performance. The initial assumption was that continuous timing, allowing a larger set of durations, would increase the number of states with significant weight and therefore have a negative performance. This effect could not be established. We do not think that this means the timing model is irrelevant. Our conclusion is rather that in the domain we consider a set of empirically observed durations indeed can be successfully approximated by a careful selection of class-specific duration models, using for instance a process such as outlined in Appendix S4.

The finding that heuristics based on goal distance improve performance supports our hypothesis in this direction. It is, however, somewhat discouraging to see that goal distance heuristics seemingly have to be very good to have a meaningful effect (cp. Sec. 3.3.3). Specifically, “intuitive recipes” seem to be essentially of no value, except when observations are very unreliable. The computation of exhaustive goal distances is clearly not possible for large state spaces. In addition, as Fig. 4 (top right) and Fig. S9 imply, exhaustive goal distances have essentially no effect when observations are already very good. From a certain perspective, this should be no surprise: All observed action sequences used more actions than strictly necessary – meaning that goal distance (at least the naive version used in this study) can not be the optimal predictor as it consistently underestimates sequence length. Eventually it points in the wrong direction and observations have to correct this. This means that further research on suitable action selection heuristics and also on the combination of different heuristics using models such as outlined in Appendix S2 are required.

As conclusion to this discussion, we have seen that all modeling aspects do have a significant and relevant impact on system performance. This means that building CSSM models which give good results in inference requires a significant amount of care in setting up the different model components. As experience with the method is gathered, this modeling process may become increasingly routine. However, it remains a major challenge for a CSSM-based approach. For the CSSM method to be accepted on a broader scale, the availability of an efficient design methodology is an important prerequisite. This needs to be addressed in future research.

### 4.4 CSSM assets

As outlined in Sec. 1.1, CSSMs are considered to have certain benefits considering reusability and flexibility. In this study, we have gained experience with two specific aspects:

**Exchange of observation models.** The *OL* observation model uses a sensor setup quite different from the *Oks* models. While the *Oks* models use the 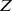-observation component (actions), the *OL* model uses the 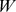 component (providing observations for the location slots of two domain objects). A switch between the two types of observation models required no change at the level of the system model. In general, a CSSM system model will accommodate any observation model that represents observations of its state variables (here we refer to the complete 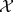 state, not just the LTS component).
As adding state variables to a CSSM model does not seem to have a negative effect (see discussion above, Sec. 4.2), CSSM methods allow to use rich state spaces that accommodate a wide variety of observation models.
**Estimating expectations on state.** Our findings concerning the estimation of state predicates (Sec. 3.4) provide anecdotal evidence to the claim that CSSM models allow to compute the expectation of arbitrary functions on state. For the predicates we used, we found a high level of estimation accuracy. This again suggests that large state spaces can efficiently be handled in CSSM models.
As for sensor models, this also means that a given CSSM model can be reused across different expectation estimation tasks, as long as the function for which to estimate the expectation can be formulated in terms of the state variables present in the CSSM model.
However, as this test has looked only at a few chosen target expectations, we do not know how reliable these estimates will be in general. In fact, we observed instances of substantial estimation errors (Fig. 9, predicate “Danger”, subject *S3*). It is not clear in how far this error is the correct estimate given the sensor data, or if it is a symptom of state space impoverishment. (As the marginal filter discards state when pruning, it may share this symptom with the particle filter.) To answer this, further research on the behavior of the marginal filter is required in order to understand how errors caused by the approximation method express themselves in the resulting estimates.

To summarize, we believe our findings regarding these two aspects indeed give some justification to the claim that CSSM models tend to provide a certain level of reusability that is not as easily accessible in methods that do not employ symbolic model definition methods. This reusability property of CSSMs is fundamentally connected with the fact that it is very easy to add state variables to a CSSM model – which on the other hand quickly creates very large state spaces. From the inferential viewpoint, large state spaces are a liability. From the viewpoint of generalizability, they are an asset. Our study suggests that it is possible to benefit from this asset without being overwhelmed by the liability it incurs.

There are two more potential assets of CSSMs that have not been considered in this study:

- Easy domain development and use of prior knowledge. CSSMs are based on symbolic representations of generative causal connections between states that describe how new states are computed from old states. It has been claimed that such generative causal structures are “more accessible to the mind” ([41], p. 21) than mere associations. This suggests that CSSMs might be particularly suited to capture expert knowledge on activity structures within a domain model. Possibly such models can even be created using only prior knowledge. However, an analysis of this topic has not been part of this study.
  On the one hand, symbolic prior knowledge has clearly influenced the underlying domain structure: domain objects, locations, state variables, and actions. On the other hand, video recordings of the test subjects' task execution have been used to identify the concrete causal structure. From our study we therefore can not conclude if it is possible to arrive at a reasonable domain model *without* any observational data. Then again it is not certain that it is mandatory to completely dispense with observation data. Already a substantial reduction in the amount of observational data required might be a significant benefit for model development. At the symbolic level already a single example allows to infer the existence of certain causal connection; it is not necessary to count frequencies.
- Reusability across different applications. This study has considered a specific application setting; investigating the reuse of individual modeling components in different application domains has not been part of the study design. While it seems reasonable that a symbolic model should provide more reusability across different application settings than a parametric subsymbolic model, our study provides no data that confirms this conjecture (or provides counter-evidence). This very interesting topic – it would arguable be the most attractive feature of CSSM-based approaches – remains an important research questions for future investigations.

### 4.5 Interleaved and erroneous actions

Our study has not explicitly considered the ability of CSSMs to cope with (or discriminate between) interleaved and erroneous actions. However, as these phenomena are a cause for considerable concerns in approaches that are based on plan libraries, such as [42], we think it is of interest to discuss briefly how this topic affects CSSM-based approaches. There are two questions: (i) can activity sequences containing erroneous actions (or actions pertaining to other goals) be reconstructed correctly and (ii) can specific actions in a reconstructed sequence be correctly labeled as erroneous or belonging to a different goal.

With respect to the first question: a CSSM is by design able to reconstruct *any* action sequence it encounters: in a given state, every action whose preconditions are met is considered as next activity – this encompasses actions that make progress towards a given goal as well as actions that move away from that goal (or that may even reach states from which it is impossible to reach the goal). Thus, since CSSMs allow any causally possible action sequence, CSSMs support the reconstruction of action sequences produced by arbitrary interleaving of composite activities and arbitrary errors. This is essentially a consequence of using explicit states (cf. Sec. 1.2 and Appendix S2).

The second question concerns how to discriminate between interleaved actions and “errors” – or, less judgmental, how to discriminate between more or less efficient actions. This is eventually based on comparing the likelihoods of two CSSM models 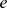 (the error model) and 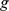 (the goal model), using the same symbolic domain model but different action selection heuristics. A less informed (or possibly misleading) selection strategy 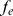 is used for the error model, the goal model uses a better informed strategy 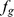 (for instance, goal distance). An action is labeled as “inefficient” or “efficient” according to which of the two models produces a higher likelihood for the action (a similar idea has been described in [43]). This also works for multiple goals, which are simply conjunctions of individual goals. Such joint goals will naturally lead to interleaved action sequences in CSSM models. The most natural way to capture this transition between different action selection regimes is to allow the 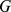 variable to vary over time (cp. Sec. 2.2.1). In the case that enumerating all potential interleaved goals is not desired or infeasible, another option is to flag actions that do not make progress towards the goal, but which do not (significantly) increase goal distance, as belonging to some interleaved activity. Again, the main challenge will be to get the heuristics right.

Incidentally, in the scenario considered in our study, the subgoals “cook meal” and “set table” allowed for a natural interleaving (which also could be found in the observation data), as well as the subgoals “not hungry” and “not thirsty”. Also, the final “clean up” sequence did provide for a substantial interleaving of different subgoals.

Since erroneous and interleaved actions are captured by CSSM approaches at the conceptual as well as the algorithmic level, we consider it legitimate to focus on the feasibility of CSSM-based inference in this study. However, we note that for a given application the concrete definition of 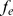 and 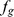 are not immediately obvious in general – our own results show that a useful definition of 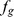 can not be reduced to intuitive recipes. Indeed, as discussed in Sec. 1.2, research on action selection strategies is an active research topic in human behavior modeling. While this is not a problem specific to CSSM-based models, but rather pertaining to all activity and intention recognition approaches, it is nevertheless a potential source of uncertainty and performance degradation.

### 4.6 Model development

Quantifying the effort spent on developing the CSSM model used in this study is not easy, as during model development also a substantial amount of method development has taken place. Assuming all methods (construction of timing models, action selection heuristics, inference engines, observation model construction) to be in place and assuming causally correct annotations to be available, CSSM model development is in fact a quite straightforward process. Building the symbolic model for the scenario used in this study by employing the iterative approach outlined in Appendix S4 took about one week for an experienced model engineer.

In contrast to this, the interleaved procedures of annotating the observation sequences, validating the causal correctness of the annotation sequences, and establishing the corresponding aLTS for these annotations took several months. One reason for this is that in the beginning comparatively inexperienced students performed the annotation process, based on an initial misjudgment of the complexity and importance of the ground truth annotation. Only half way through the annotation process we became aware of the fact that even in seemingly well understood everyday domains the production of causally correct annotations requires careful ontology design and is, in fact, that process which produces the main bulk of insight into the causal structure of the domain. It is therefore, in hindsight, quite surprising that in current research on activity and intention recognition the process of getting the annotations right is usually absent from the method description. Indeed, we found other publicly available sets of observation data on everyday activities – such as [31] – as lacking in annotation correctness as our own initial attempts. While there is some work on annotation methodology [29], [44], [45], none is concerned with causally correct annotations. We think that research domains such as content analysis and business process analysis, which are also concerned with reliably and reproducibly annotating real world phenomena with a set of formal notations, might provide valuable tools and methods for creating dependable annotations (see for instance [46] and [47]).

Considering the iLTS model, we found anecdotal evidence that (inexperienced) model engineers are prone to modeling errors such as deadlocks, livelocks, and similar phenomena. The possibility of such errors to occur is a natural consequence of the innate capabilities of CSSMs to represent the interleaved execution of multiple threads of activity. In such settings, locks are typically used to synchronize threads [48]. Getting lock management wrong then results in deadlocks or livelocks (or multiple persons sitting on the same chair). We note that the iLTS model produces 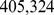 states with infinite goal distance, i. e. livelocks: regions in the iLTS graph that can be entered from the initial state, but which provide no success state and no exit. It therefore seems advisable to employ established methods of software engineering such as code analysis [49] and model checking [50] to avoid at least the detectable modeling errors from the iLTS model.

As discussed in Sec. 4.3, the success of CSSM-based inference depends on all modeling factors, not only the iLTS model. In so far, the use of CSSMs raises a new challenge for model engineering, as they combine symbolic, algorithmic modeling aspects with probabilistic and statistical reasoning. In developing CSSM models, it is necessary to understand both domains of modeling, as well as the interactions between them.

### 4.7 Limitations of the study

Performance values (such as accuracy) have been reported for comparison of different models *within* this study. We do not consider them as indicators for prospective performance at an *absolute* level for the following reasons:

- The observation models have used a very simple approach; no detailed modeling of, for instance, motion trajectories has been attempted. Therefore, performance results may be overly pessimistic. On the other hand, use of scrambling to make sure observations fulfill the i.i.d. property is not available for real world settings. This may lead to performance to be too optimistic.
  As baseline and CSSM equally profit from both effects, model comparisons should be valid. But it is not clear in how far the absolute performance is biased.
- As explained in Sec. 2.1.2, practical methodological constraints have inhibited the use of procedures for establishing reliable absolute performance results, such as cross-validation. This means the performance data we used for comparison purposes *within* this study can not be used for inferring statements on potential absolute performance or for comparison with performance results reported in other studies. (However, considering the substantial variance in experimental procedures between different studies, we doubt that a meaningful comparison would have been possible anyway.)

As discussed previously, neither the performance in discriminating between different goals was considered in this study nor the ability to detect erroneous actions or interleaved activities. Although the observed action sequences obviously contained suboptimal actions (all observed action sequences were substantially longer than the goal distance computed for the starting state), no attempt was made to flag individual actions in the estimated sequence as “efficient” or “inefficient”. Also, although there were interleaved composite activities (setting the table, cooking the meal), it was not attempted to label the estimated action with the composite activity they potentially belong to.

These questions, as well as the discrimination between different goals, can most naturally be solved by appropriate use of the 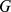 variable (cf. Sec. 4.5 and 2.2.1): different values of 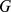 select different action selection regimes, so that from the observed actions the most probable 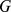 can be estimated via its effect on the action selection 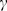. While this is conceptually straightforward and the appropriate approach, this study provides no data that allows a statement on the practical viability and the performance to expect.

A further limitation concerns the coarse-grained estimation target (16 classes), which is effectively a consequence of the baseline comparison approach and the low-resolution observation model (cf. Sec. 2.4.2). The *Oks* models only allow to discriminate between action classes. It is therefore impossible to reconstruct, for instance, the exact sequence in which 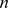 items have been cleaned from the observation of 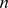 “wash” actions. Any permutation is possible given the action selection heuristics and observations. This means the model used in this study provides a level of detail that is not resolvable by observation models. From the viewpoint of model reusability (see Sec. 4.8 below), this “latent detail” may be actually desirable. However, this study gives no information on the question whether latent detail indeed can be made accessible by other observation models. (In comparison to previous research, where a median number of 6 target classes has been used, see Sec. 1.3.2, the 16 targets in this study are in fact quite fine grained.)

Finally, this study focuses on a very specific application domain – human activity recognition. Concerning the CSSM method, this may induce a unnecessary conceptual restriction, as the method is applicable to sequential state estimation in any non-deterministic dynamic system that lends itself to a symbolic description.

### 4.8 Conclusion

CSSMs are a conceptually interesting approach to defining state space models for sequential state estimation in dynamic systems, where the system model is built from symbolic prior knowledge on the causal – computational – structure of actions. An interesting application domain for this approach is tracking human activities. The use of this approach makes it easy to define very detailed state models, which produce very large, usually categorical state spaces. Concerning prior research, it is not clear whether successful inference in these environments is possible outside of very simple scenarios. Existing methods for non-linear non-Gaussian system dynamics, such as particle filters, may be incompatible with the non-metric structure of the state space.

In this paper, we have presented a unified view on the different instances of the CSSM method, based on the definition of latently infinite LTS using computational action languages and a probabilistic semantics for the resulting model. We regard this in itself as an important contribution, as it should help researchers to relate the different approaches under this common view.

The results of the empirical study imply that scenarios may be easily several orders of magnitude larger than have been considered in existing studies on CSSM. Our results further indicate that it is indeed possible to use the resulting models for inference without compromising performance in comparison to typical systems built from training data. This is encouraging for further research on CSSMs as it shows that the method is potentially applicable outside the limitations of confined laboratory experiments. We have also shown that in order to achieve this performance, a range of modeling parameters has to be carefully adjusted, as all of these parameters have a significant and substantial impact on inference performance. This is a very important result, as these effects have so far not been discussed in research on CSSMs. (One explanation for this is that in the limited scenarios investigated so far the effect of these factors has simply been not significant and therefore been overlooked by the investigators.) Specifically, we have found sequential Monte-Carlo methods (i. e., particle filter) to be not suitable for approximate inference in CSSMs; a fact that has not been discussed in present CSSM research (again, this may be due to the effect being not visible in small scenarios). Instead we propose use of a marginal filter, which previously has been shown to improve performance in state estimation based on plan libraries [8].

In addition to these results, the study provides a blueprint for a systematic CSSM model construction that should allow other researchers to reproduce our results and to apply the CSSM method systematically to other scenarios.

Nevertheless, this study is only a first step towards making CSSMs and latently infinite LTS applicable to real world activity recognition settings. The following points seem to be of prime importance as next steps:

- Improving observation model and system model.
  As shown in Sec. 3.3.1, choice of observation model had the strongest impact on performance. Considering the rather simple approach used in this study, the obvious consequence is to experiment with more refined observation models. Dense observations [19] as well a more detailed modeling of motion trajectories are immediate options; as is the use of camera-based tracking. The first step should be to ensure i.i.d. observations without requiring to resort to scrambling as this clearly is infeasible in real-world applications. The next step is to analyze in how far additional model detail becomes resolvable through the use of improved observations.
  With respect to the system model, it is important to understand which effectively computable action selection heuristics (having the second largest effect) are really helpful in improving performance.
- Exploiting 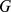 for intention recognition and error detection.
  As discussed in Sec. 4.7, the conceptual approach is straightforward. Indeed, for laboratory examples this method has already been shown to work [1], [ 5]. Objective of a further study on this question should be to investigate how to apply this method at the practical level using a state space of realistic complexity and how large goal sets can be handled.
- Analyze model construction methodology and use of prior knowledge.
  A strong point claimed for CSSMs is the ability to replace training data by prior knowledge. So far, it has not been shown conclusively that this assumption really holds – there has been no study looking at this question. In order to assess the cost and benefit of CSSM-based approaches to model construction in comparison to other methods, it is necessary to understand which amount of training data and which amount of annotation effort really can be replaced by prior knowledge.
  In this context it is also of interest to analyze in how far symbolic models can be reconstructed from activity traces, a topic considered in process mining [51], learning of planning domains [52], and also in web mining [53]. While still requiring training data, such methods possibly can produce candidate models for review, refinement, and generalization by a modeling expert, thereby simplifying the task of model construction.
- Analyze model reusability. Eventually, exchange of observation models, flexible state predicates, plan-synthesis, and rich state spaces are all aspects of model reusability. It needs to be shown that these reusability properties potentially provided by CSSMs – which we have found to exist in two instances – are indeed present in general and of practical relevance.

When addressing these questions, it may become necessary to further improve the implementation of the inference method in order to keep inference tractable. This might include a more efficient representation of states (using, e.g. binary decision diagrams [50] instead of tries as dictionaries, see Sec. 4.2.1 of Appendix S4), the support of continuous domains (employing Rao-Blackwellization [54]), or the use of approximate state representations, for instance by ignoring higher order interactions [55].

## Supporting Information

Figure S1 **Physical setup of experiment.** Left: Participant sensor instrumentation. Right: Stage and props used in experiment.
(TIFF)

Figure S2
**Effect of scrambling on (expected) log probability of observations vs. normalized relative run position.** Red lines are computed by locally weighted regression using the loess function in R. Right plot: detail of left plot. Consider a run of 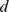 observations 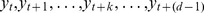, all labeled with class 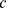. The relative position of observation 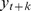 in this run is 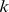. Relative positions range from 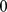 to 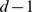. The *normalized* relative position of observation 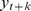 is 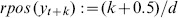. Relative run positions are values between 0 and 1. Adding the term 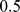 puts the normalized relative position of the run's center (with relative position at 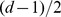) at 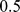. The figure shows a scatterplot of the log probabilities of the given observations (using the model 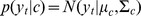) versus their normalized relative run positions. The local regression curves, representing approximations to the expected values, show the centering effect proposed in Sec. 4.1.3 of Appendix S4. (A preliminary analysis of individual actions suggests this effect to be more prominent in actions with longer duration, in agreement with this hypothesis.). The figure suggests that there is an influence of the squared distance between an observations relative position and the run center, given by 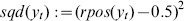, and the expected probability. This is indeed the case: a linear model for predicting 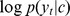 from 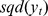 shows a highly significant influence (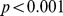), while this influence can not be established for scrambled data (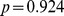). However, 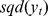 explains only 2.2% in 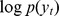 variance – it is therefore quite interesting that scrambling has such a massive influence on accuracy (this could possibly be due to the cumulative effect – all observations at the start of a run are affected –, and the fact that a few percentage points in logarithmic scale may represent a factor of two or more in linear scale).(TIFF)

Figure S3
**Frequencies of action classes in empirical data.** *WASH* is the most frequent activity with a proportion of 19.8%, giving an uninformed baseline accuracy of 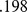.(TIFF)

Figure S4
**Preprocessed observations (principal components factor loadings) and ground truth.** The black line shows the ground truth (action class, 16 classes) for each subject. The heatmap gives the factor loadings for the 21 prinicpal components used in *O21*, one component per row (color coding: 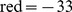, 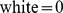, 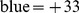).(TIFF)

Figure S5 **Scaled confusion matrices for different inference methods.** Columns sum to one, diagonal thus gives sensitivity. Class labels sorted by number of action instances for class.
(TIFF)

Figure S6 **Details for action class estimates produced by baseline classifiers and CSSM models.** Comparison of the action class estimate for the different classifier grouped by subject. On the left side the results of the QDA, the forward filtered HMM (HMMf) and marginal filter (CMf) are illustrated. The right side gives the MAP (CMv) sequence as well as the smoothed estimates of HMM (HMMs) and marginal filter (CMs).
(TIFF)

Figure S7
**Comparison of different performance metrics.** Each box shows a different distance measure, within a box the specific measure is used to compare the different *Mode* factors. The performance gives the distance relative to the worst inference method (always QDA), smaller means better. The figure provides the results of different performance metrics. Levenshtein edit distance has unit cost for insertion, deletion, and substitution operations. 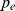 is the error probability, given by 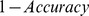. 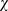 is the difference in numbers of actions, given by 
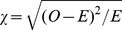
 where 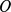 is the observed number of actions (the number of actions in the estimation sequence) and 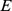 the expected number of actions (as given by the ground truth). 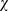 represents the intuition that out-of-sequence actions, as discussed in Sec. 2.4.2, will increase the number of runs in the estimated action sequence. 
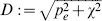
 is an Euclidian distance interpretation of 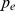 and 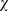. DTW is the dynamic time warping distance, computed based on a 0-1-distance for sequence elements. In the upper row of the figure the values of each metric 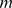 have been divided by the maximum value for 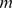, in the lower row the values for each subject 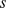 in each metric 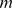 have been divided by the maximum value for 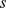 in 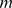. To allow a better assessment of the separation of the different *Method* levels afforded by these measures, data points for different subjects within a method have been connected by lines. In order to visually untangle the resulting plots, subjects have been sorted be mean DTW value. D and DTW give a clearer separation of the four groups 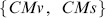, 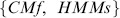, 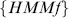, and 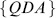 than is given by 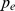 resp. Levenshtein distance. Specifically, in DTW *CMf* performs at the same level as *HMMs*. In other words: if the temporal structure is taken into account for performance evaluation, by using measures such as D or DTW, the performance advantage of CSSMs over HMMs is even more prominent than indicated by accuracy alone.(TIFF)

Figure S8
**Complete accuracy data for 432 CSSM model configurations.** Details for the *O21s* configurations marked by triangles are shown in Fig. 5. The orange triangles mark the *CM* configurations used in testing 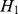.(TIFF)

Figure S9 **Interactions between** *Observations*, *Distance*, **and** *Weight*.
(TIFF)

Figure S10 **Interactions between** *Mode*, *Distance*, **and** *Weight*.
(TIFF)

Figure S11
**Distribution of median relative state counts in filtering runs.** Left and center plots: empirical distribution function and density estimate of iLTS states (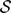 states) per unit (SpU). Right plot: empirical distribution function of inference states (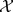 states) per unit (XpU).(TIFF)

Figure S12
**Jensen Shannon distance and accuracies for different values for** *Target* **and** *Distance*. Plots are based on configuration 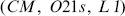. Details for the bottom right configuration 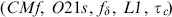 are shown in Fig. 9.(TIFF)

Table S1
**Task script and** 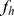 **distance values.**
(PDF)

Table S2
**Basic properties of observed action sequences.** *Steps*: number of actions in sequence (641 in total), *Plan.Length* (549 in total) ignores actions without effect (“wait”). *Frames*, *Duration*: sequence duration in frames (6647 in total) and seconds. 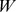, 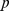: statistic and 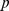-value for Shapiro-Wilk normality test (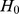  =  data has normal distribution, 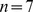). There is no significant correlation between temporal length of an action sequence (*Duration*) and the number of action it contains (*Steps*) (Pearson's 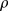, 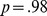). Use of Pearson's 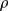 justified by normality of data, cf. results of Shapiro-Wilk tests.(PDF)

Table S3 **Action sequence of subject** ***S1*** **(aLTS annotations).**
(PDF)

Table S4
**Domain objects and slots.** The domain objects and their slots. All slots have boolean value domains with two exceptions:
*available(hands)* is an integer. The actions implement the constraint 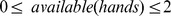.
*location(* 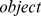 *)* is a symbolic value. Allowed values for the different objects are given in Table S5 (these constraints are again implemented by the actions).
(PDF)

Table S5 **Value domains of** ***location*** **slot by domain object.** (1152000 potential combinations).
(PDF)

Table S6
**Finding the distribution for pooled action durations.** Finding the distribution for pooled action durations (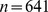). LL  =  log-likelihood of observed durations under corresponding parametric model. 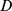 and 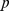 are statistic and *p*-value for the Kolmogorov-Smirnov test with the null that observed data has been drawn from parametric distribution. (As the parameters have been estimated from the sample data, the assumptions of the KS test are violated. Nevertheless the *p*-values obtained agree with the log-likelihood.).(PDF)

Table S7 **Duration models selected for action classes.**
(PDF)

Table S8
**Comparing performance of different CSSM model configurations to HMM model.** Comparing performance of different CSSM model configurations to HMM model, using paired 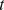-test and Wilcoxon signed rank tests (CM, O21s, L1). For all 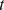-tests, 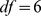. 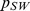 gives the p-value for the Shapiro-Wilk normality test.(PDF)

Table S9 **Significance of effects of CSSM configuration factors on** ***Accuracy.*** Significance of effects of CSSM configuration factors on *Accuracy*, using 216 *CMf*/*CPf* configurations. (2 modes, 3 observations, 3 distances, 6 weights, 2 durations.).
(PDF)

Data S1 **Observation model and pre-processed sensor data.**
(ZIP)

Appendix S1 **Notational conventions and abbreviations.**
(PDF)

Appendix S2 **Computational state space models.**
(PDF)

Appendix S3 **Annotation procedure and annotation ontology.**
(PDF)

Appendix S4 **Detailed model description.**
(PDF)
