# Supplementary figures and images for: Computational State Space Models for Activity and Intention Recognition. A Feasibility Study
Source: PLoS One. 2014 Nov 5;9(11):e109381. doi: 10.1371/journal.pone.0109381 (PMC4220990; doi:10.1371/journal.pone.0109381)

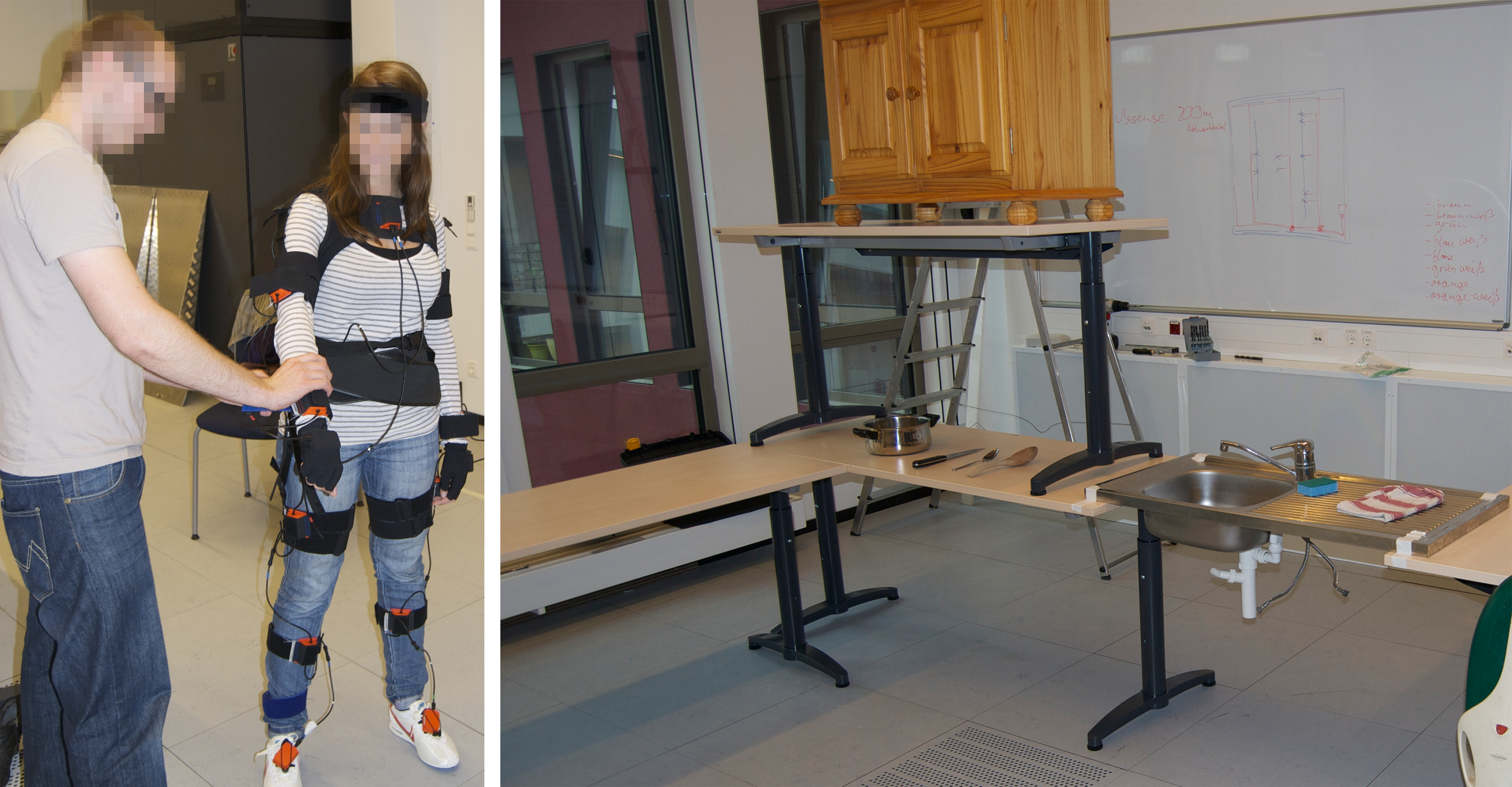

Supplement: Figure S1 — Physical setup of experiment. Left: Participant sensor instrumentation. Right: Stage and props used in experiment. (TIFF) [file pone.0109381.s001.tiff]

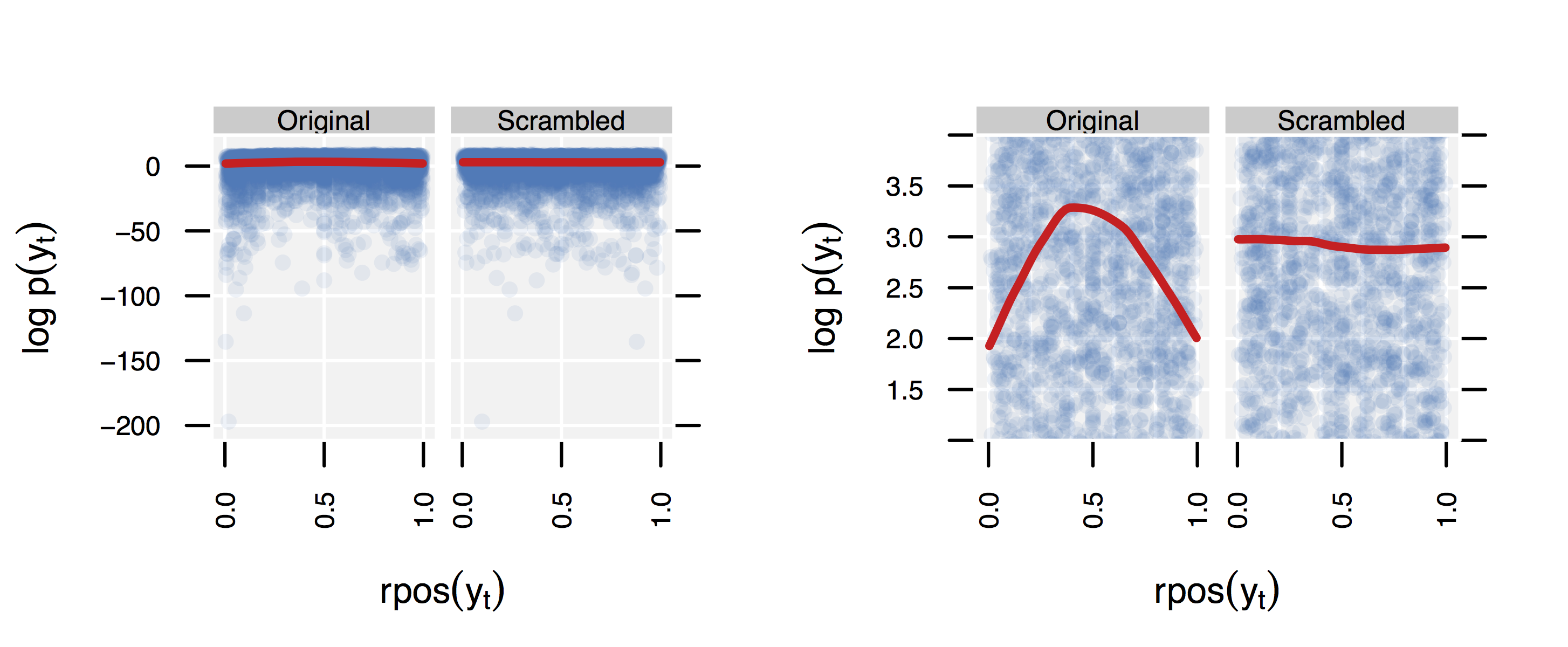

Supplement: Figure S2 — Effect of scrambling on (expected) log probability of observations vs. normalized relative run position. Red lines are computed by locally weighted regression using the loess function in R. Right plot: detail of left plot. Consider a run of observations , all labeled with class . The relative position of observation in this run is . Relative positions range from to . The normalized relative position of observation is . Relative run positions are values between 0 and 1. Adding the term puts the normalized relative position of the run's center (with relative position at ) at . The figure shows a scatterplot of the log probabilities of the given observations (using the model ) versus their normalized relative run positions. The local regression curves, representing approximations to the expected values, show the centering effect proposed in Sec. 4.1.3 of Appendix S4. (A preliminary analysis of individual actions suggests this effect to be more prominent in actions with longer duration, in agreement with this hypothesis.). The figure suggests that there is an influence of the squared distance between an observations relative position and the run center, given by , and the expected probability. This is indeed the case: a linear model for predicting from shows a highly significant influence (), while this influence can not be established for scrambled data (). However, explains only 2.2% in variance – it is therefore quite interesting that scrambling has such a massive influence on accuracy (this could possibly be due to the cumulative effect – all observations at the start of a run are affected –, and the fact that a few percentage points in logarithmic scale may represent a factor of two or more in linear scale). (TIFF) [file pone.0109381.s002.tiff]

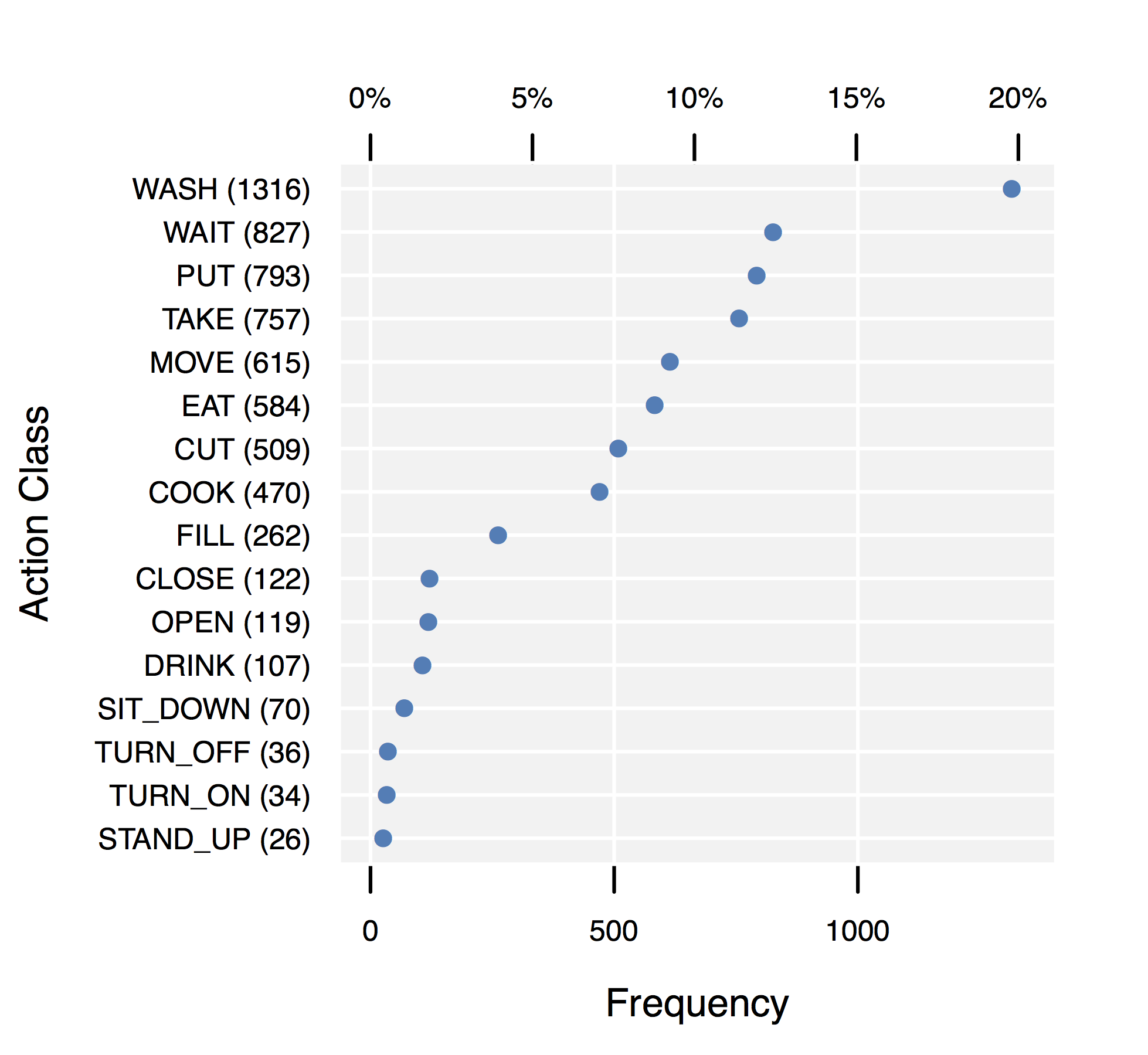

Supplement: Figure S3 — Frequencies of action classes in empirical data. WASH is the most frequent activity with a proportion of 19.8%, giving an uninformed baseline accuracy of . (TIFF) [file pone.0109381.s003.tiff]

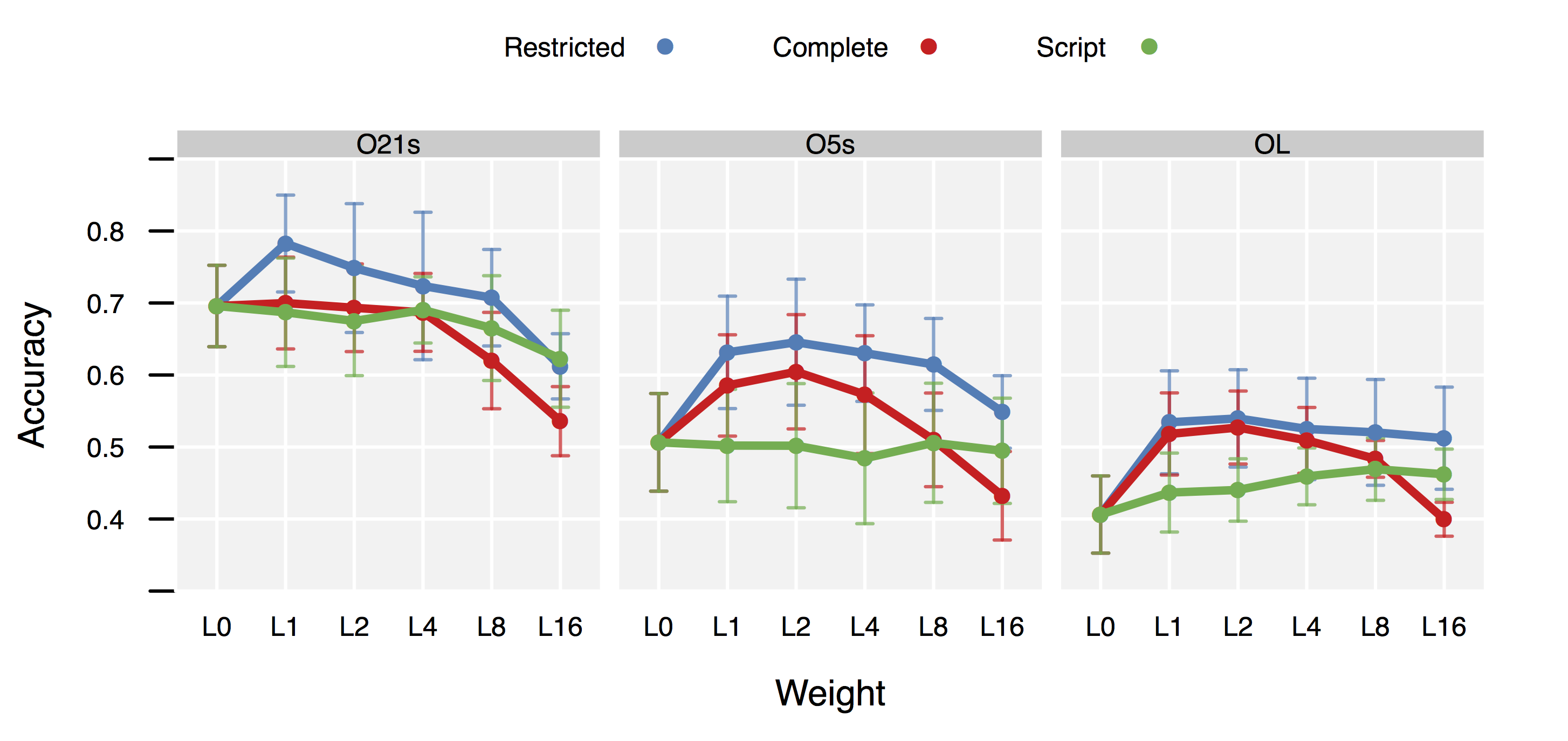

Supplement: Figure S9 — Interactions between Observations, Distance, and Weight. (TIFF) [file pone.0109381.s009.tiff]

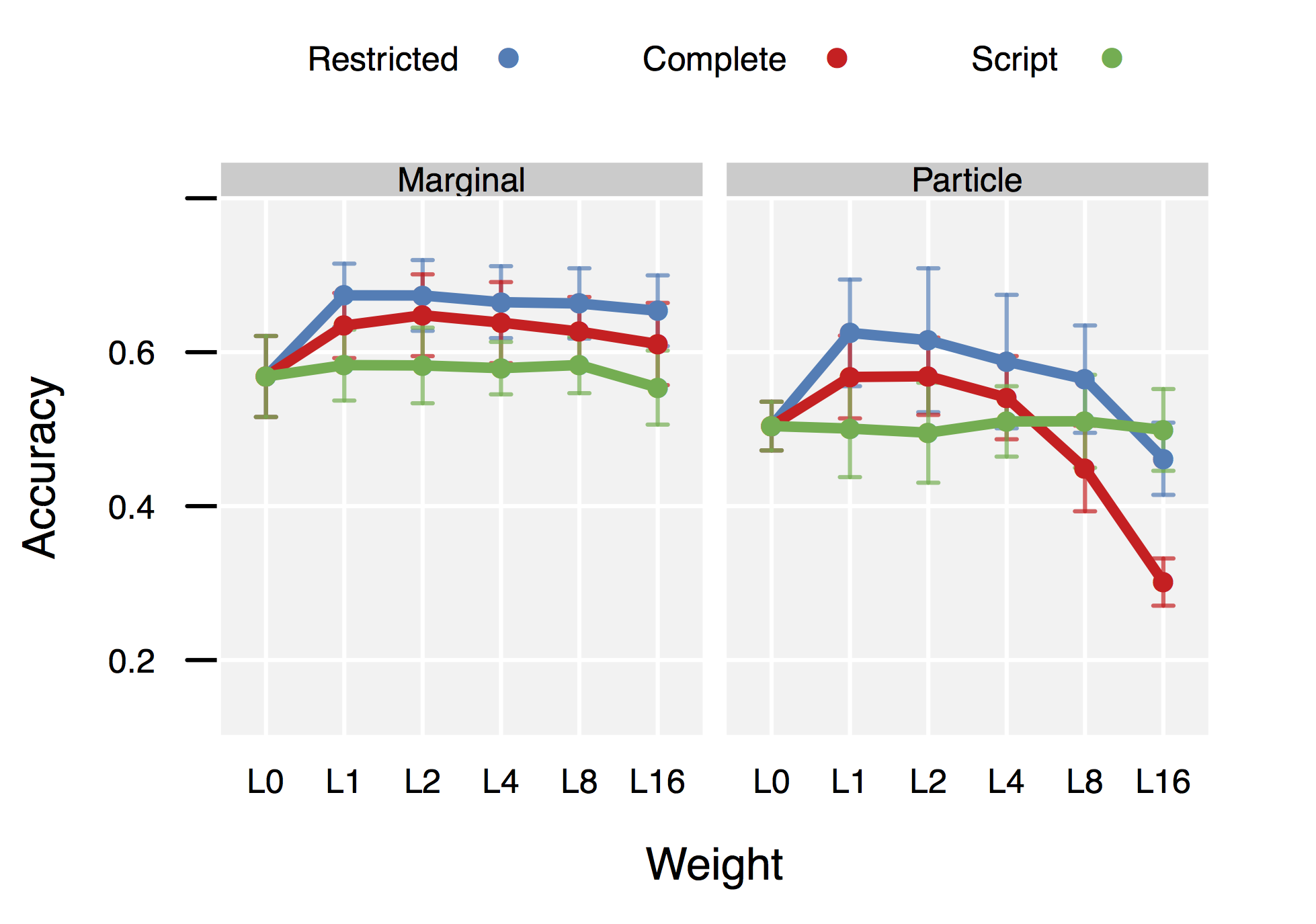

Supplement: Figure S10 — Interactions between Mode, Distance, and Weight. (TIFF) [file pone.0109381.s010.tiff]
