# Supplementary material for: Computational State Space Models for Activity and Intention Recognition. A Feasibility Study
Source: PLoS One. 2014 Nov 5;9(11):e109381. doi: 10.1371/journal.pone.0109381 (PMC4220990; doi:10.1371/journal.pone.0109381)
Supplement: Table S1 — Task script and distance values. (PDF) [file pone.0109381.s013.pdf]

Table S1. Task script and f_h distance values.

Step	f_h	Task	State predicate if task not fulfilled
1	14	Clean hands	– (no state predicate with lower step count matches)
2	13	Get food to sink	not clean food, holds food, not at sink
3	12	Clean food	not clean food, holds food, at sink
4	11	Cut food	clean food, not food prepared
5	10	Turn stove on	food prepared, not cooked, stove off
6	9	Cook food	food prepared, not cooked, stove on
7	8	Turn oven off	hungry, cooked, stove on
8	7	Finish setting table & Sit down	hungry, cooked, stove off, not seated
9	6	Enjoy meal	hungry, stove off, seated
10	5	Get up	not hungry, seated
11	4	clean kitchen utensil (ku)	not hungry, not seated, 4 ku dirty
12	3	clean ku	not hungry, not seated, 3 ku dirty
13	2	clean ku	not hungry, not seated, 2 ku dirty
14	1	clean ku	not hungry, not seated, 1 ku dirty
15	0	done	
