# Supplementary material for: Computational State Space Models for Activity and Intention Recognition. A Feasibility Study
Source: PLoS One. 2014 Nov 5;9(11):e109381. doi: 10.1371/journal.pone.0109381 (PMC4220990; doi:10.1371/journal.pone.0109381)
Supplement: Table S2 — Basic properties of observed action sequences. Steps: number of actions in sequence (641 in total), Plan.Length (549 in total) ignores actions without effect (“wait”). Frames, Duration: sequence duration in frames (6647 in total) and seconds. , : statistic and -value for Shapiro-Wilk normality test ( = data has normal distribution, ). There is no significant correlation between temporal length of an action sequence (Duration) and the number of action it contains (Steps) (Pearson's , ). Use of Pearson's justified by normality of data, cf. results of Shapiro-Wilk tests. (PDF) [file pone.0109381.s014.pdf]

Table S2. Basic properties of observed action sequences.

	S1	S2	S3	S4	S5	S6	S7	*M*	*SD*	*W*	*p*
Steps	89	91	96	96	82	95	92	91.6	5.00	0.87	0.18
Plan.Length	77	77	79	87	66	82	81	78.4	6.48	0.91	0.42
Frames	1028	903	1032	634	909	1205	936	949.6	174.06	0.93	0.57
Duration	274	241	275	169	242	321	250	253.2	46.42	0.93	0.57

Steps: number of actions in sequence (641 in total), Plan.Length (549 in total) ignores actions without effect ("wait"). Frames, Duration: sequence duration in frames (6647 in total) and seconds. W, p: statistic and p-value for Shapiro-Wilk normality test (H_0 = data has normal distribution, $n = 7$). There is no significant correlation between temporal length of an action sequence (Duration) and the number of action it contains (Steps) (Pearson's ρ, $p = .98$). Use of Pearson's ρ justified by normality of data, cf. results of Shapiro-Wilk tests.
