# Supplementary material for: Computational State Space Models for Activity and Intention Recognition. A Feasibility Study
Source: PLoS One. 2014 Nov 5;9(11):e109381. doi: 10.1371/journal.pone.0109381 (PMC4220990; doi:10.1371/journal.pone.0109381)
Supplement: Table S3 — Action sequence of subject S1 (aLTS annotations). (PDF) [file pone.0109381.s015.pdf]

Table S3. Action sequence of subject S1 (aLTS annotations).

Step	Time	Action	Step	Time	Action
1	0	wash hands	46	511	sit-down
2	35	wait	47	536	take spoon plate
3	43	move sink counter	48	541	eat
4	64	take food counter	49	616	put spoon plate
5	70	move counter sink	50	619	take glass table
6	77	wash food	51	624	drink
7	111	move sink counter	52	631	wait
8	117	take knife counter	53	638	drink
9	122	put food cutting-board	54	647	put glass table
10	126	cut food	55	653	take spoon plate
11	173	put knife counter	56	657	eat
12	177	wait	57	692	put spoon plate
13	184	take knife+cutting-board counter	58	699	take plate+glass table
14	191	fill food cutting-board pot	59	705	stand-up
15	204	put knife+cutting-board counter	60	710	move table sink
16	211	take pot counter	61	724	wait
17	219	put pot stove	62	729	put glass sink
18	228	wait	63	735	wait
19	230	turn-on stove	64	759	put plate sink
20	235	wait	65	767	take sponge sink
21	247	take wooden-spoon counter	66	773	take spoon plate
22	256	cook	67	777	wash spoon
23	334	put wooden-spoon pot	68	797	wait
24	336	turn-off stove	69	809	wash spoon
25	341	wait	70	816	put spoon sink
26	349	open cupboard	71	822	take plate sink
27	360	take plate+glass cupboard	72	825	wash plate
28	376	put plate+glass counter	73	874	put plate sink
29	389	take pot stove	74	881	take glass sink
30	395	fill food pot plate	75	884	wash glass
31	416	put pot stove	76	919	put glass sink
32	422	wait	77	926	put sponge sink
33	427	take bottle counter	78	929	move sink counter
34	433	open bottle	79	935	take pot stove
35	441	fill water bottle glass	80	939	move counter sink
36	453	close bottle	81	943	put pot sink
37	463	put bottle counter	82	946	take wooden-spoon pot
38	467	wait	83	948	take sponge sink
39	469	take plate counter	84	950	wash wooden-spoon
40	474	wait	85	964	put wooden-spoon sink
41	481	take spoon counter	86	970	take pot sink
42	485	put spoon plate	87	973	wash pot
43	488	take glass counter	88	1020	put pot sink
44	491	move counter table	89	1027	put sponge sink
45	506	put plate+glass table	90	1028	(done)
