# Supplementary material for: Computational State Space Models for Activity and Intention Recognition. A Feasibility Study
Source: PLoS One. 2014 Nov 5;9(11):e109381. doi: 10.1371/journal.pone.0109381 (PMC4220990; doi:10.1371/journal.pone.0109381)
Supplement: Table S4 — Domain objects and slots. The domain objects and their slots. All slots have boolean value domains with two exceptions: available(hands) is an integer. The actions implement the constraint . location( ) is a symbolic value. Allowed values for the different objects are given in Table S5 (these constraints are again implemented by the actions). (PDF) [file pone.0109381.s016.pdf]

Table S4. Domain objects and slots

	available	clean	cooked	filled	hungry	location	on	open	prepared	seated	thirsty
bottle						•		•			
cupboard								•			
cutting_board						•					
food		•	•			•			•		
glass		•		•		•					
hands	•	•									
knife						•					
plate		•				•					
pot		•				•					
self					•	•				•	•
sponge						•					
spoon		•				•					
stir_spoon		•				•					
stove							•				

The domain objects and their slots are given in Tbl. S4. All slots have boolean value domains with two exceptions:

- available(hands) is an integer. The actions implement the constraint $0 \leq$ available(hands) ≤ 2.
- location(*object*) is a symbolic value. Allowed values for the different objects are given in Tbl. S5 (these constraints are again implemented by the actions).
