# Supplementary material for: Computational State Space Models for Activity and Intention Recognition. A Feasibility Study
Source: PLoS One. 2014 Nov 5;9(11):e109381. doi: 10.1371/journal.pone.0109381 (PMC4220990; doi:10.1371/journal.pone.0109381)
Supplement: Table S5 — Value domains of location slot by domain object. (1152000 potential combinations). (PDF) [file pone.0109381.s017.pdf]

Table S5. Value domains of location slot by domain object. (1152000 potential combinations)

	counter	cupboard	cutting_board	hands	plate	stove	pot	sink	table	void	# locations
bottle	•			•							2
cutting_board	•			•							2
food	•		•	•	•		•			•	6
glass	•	•		•				•	•		5
knife	•			•				•	•		4
plate	•	•		•				•	•		5
pot	•			•		•		•			4
self	•							•	•		3
stir_spoon	•			•			•	•			4
sponge				•				•			2
spoon	•			•	•			•	•		5
