# Supplementary material for: Computational State Space Models for Activity and Intention Recognition. A Feasibility Study
Source: PLoS One. 2014 Nov 5;9(11):e109381. doi: 10.1371/journal.pone.0109381 (PMC4220990; doi:10.1371/journal.pone.0109381)
Supplement: Table S6 — Finding the distribution for pooled action durations. Finding the distribution for pooled action durations (). LL = log-likelihood of observed durations under corresponding parametric model. and are statistic and p-value for the Kolmogorov-Smirnov test with the null that observed data has been drawn from parametric distribution. (As the parameters have been estimated from the sample data, the assumptions of the KS test are violated. Nevertheless the p-values obtained agree with the log-likelihood.). (PDF) [file pone.0109381.s018.pdf]

Table S6. Finding the distribution for pooled action durations.

Model	LL	D	p
cauchy	−2137.01	0.20	0
exponential	−2144.55	0.16	5.22×10^{-15}
gamma	−2137.04	0.17	9.99×10^{-16}
geometric	−2147.16	0.23	0
lognormal	−2011.15	0.10	1.47×10^{-5}
negative binomial	−2164.15	0.18	0
normal	−2672.07	0.28	0
Poisson	−4988.42	0.44	0
weibull	−2144.44	0.17	8.88×10^{-16}

Finding the distribution for pooled action durations ($n = 641$). LL = log-likelihood of observed durations under corresponding parametric model. D and p are statistic and p-value for the Kolmogorov-Smirnov test with the null that observed data has been drawn from parametric distribution. (As the parameters have been estimated from the sample data, the assumptions of the KS test are violated. Nevertheless the p-values obtained agree with the log-likelihood.)
