# Supplementary material for: Computational State Space Models for Activity and Intention Recognition. A Feasibility Study
Source: PLoS One. 2014 Nov 5;9(11):e109381. doi: 10.1371/journal.pone.0109381 (PMC4220990; doi:10.1371/journal.pone.0109381)
Supplement: Table S7 — Duration models selected for action classes. (PDF) [file pone.0109381.s019.pdf]

**Table S7.** Duration models selected for action classes

| Class | $n$ | Model | LL | $D$ | $p$ |
|---|---|---|---|---|---|
| Base | 143 | lognormal | −410.61 | 0.07 | 0.5254 |
| COOK | 9 | weibull | −44.10 | 0.27 | 0.4367 |
| CUT | 7 | gamma | −32.36 | 0.18 | 0.9512 |
| DRINK | 11 | weibull | −21.55 | 0.21 | 0.6531 |
| EAT | 13 | lognormal | −59.49 | 0.20 | 0.5962 |
| FILL | 21 | weibull | −62.79 | 0.19 | 0.4025 |
| MOVE | 54 | lognormal | −162.58 | 0.08 | 0.8349 |
| PUT | 162 | lognormal | −355.67 | 0.15 | 0.0015 |
| STAND_UP | 7 | weibull | −10.01 | 0.19 | 0.9153 |
| TAKE | 165 | lognormal | −358.01 | 0.14 | 0.0027 |
| WASH | 49 | gamma | −207.42 | 0.08 | 0.8985 |
