# Supplementary material for: Computational State Space Models for Activity and Intention Recognition. A Feasibility Study
Source: PLoS One. 2014 Nov 5;9(11):e109381. doi: 10.1371/journal.pone.0109381 (PMC4220990; doi:10.1371/journal.pone.0109381)
Supplement: Table S8 — Comparing performance of different CSSM model configurations to HMM model. Comparing performance of different CSSM model configurations to HMM model, using paired -test and Wilcoxon signed rank tests (CM, O21s, L1). For all -tests, . gives the p-value for the Shapiro-Wilk normality test. (PDF) [file pone.0109381.s020.pdf]

Table S8. Comparing performance of different CSSM model configurations to HMM model.

Duration	Distance	Mode	t	p_t	M_t	V	p_V	M_V	p_{SW}
τ_c (continuous)	f_δ (complete)	Forward	3.14	.02	.037	27	.031	.036	.043
τ_d (discrete)	f_δ (complete)	Forward	7.87	$<$ .001	.067	28	.016	.07	.14
τ_c (continuous)	$f_{\bar{\delta}}$ (restricted)	Forward	5.78	.001	.066	28	.016	.069	.12
τ_d (discrete)	$f_{\bar{\delta}}$ (restricted)	Forward	9.37	$<$ .001	.098	28	.016	.11	.081
τ_c (continuous)	f_h (script)	Forward	3.61	.011	.043	27	.031	.042	.43
τ_d (discrete)	f_h (script)	Forward	7.67	$<$ .001	.064	28	.016	.064	.79
τ_c (continuous)	f_δ (complete)	Smoothing	6.75	$<$ .001	.063	28	.016	.068	.078
τ_d (discrete)	f_δ (complete)	Smoothing	6.70	$<$ .001	.083	28	.016	.087	.12
τ_c (continuous)	$f_{\bar{\delta}}$ (restricted)	Smoothing	6.71	$<$ .001	.082	28	.016	.087	.16
τ_d (discrete)	$f_{\bar{\delta}}$ (restricted)	Smoothing	5.88	.001	.1	28	.016	.11	.27
τ_c (continuous)	f_h (script)	Smoothing	7.14	$<$ .001	.06	28	.016	.064	.12
τ_d (discrete)	f_h (script)	Smoothing	6.27	$<$ .001	.08	28	.016	.092	.044

Comparing performance of different CSSM model configurations to HMM model, using paired t-test and Wilcoxon signed rank tests (CM, O21s, L1). For all t-tests, $df = 6$. p_{SW} gives the p-value for the Shapiro-Wilk normality test.
