# Supplementary material for: Computational State Space Models for Activity and Intention Recognition. A Feasibility Study
Source: PLoS One. 2014 Nov 5;9(11):e109381. doi: 10.1371/journal.pone.0109381 (PMC4220990; doi:10.1371/journal.pone.0109381)
Supplement: Table S9 — Significance of effects of CSSM configuration factors on Accuracy. Significance of effects of CSSM configuration factors on Accuracy, using 216 CMf/CPf configurations. (2 modes, 3 observations, 3 distances, 6 weights, 2 durations.). (PDF) [file pone.0109381.s021.pdf]

Table S9. Significance of effects of CSSM configuration factors on Accuracy

Effect	F value		p value		η^2_G	
Mode	$F_{(1,6)}$	= 168.93	< .001	*	.34	*
Observations	$F_{(2,12)}$	= 34.51	< .001	*	.61	*
Distance	$F_{(2,12)}$	= 23.23	< .001	*	.14	*
Weight	$F_{(5,30)}$	= 39.25	< .001	*	.18	*
Duration	$F_{(1,6)}$	= 1.76	.23		.004	
Mode:Observations	$F_{(2,12)}$	= 79.17	< .001	*	.24	*
Mode:Distance	$F_{(2,12)}$	= 13.68	< .001	*	.033	
Observations:Distance	$F_{(4,24)}$	= 3.34	.026	*	.03	
Mode:Weight	$F_{(5,30)}$	= 19.65	< .001	*	.089	*
Observations:Weight	$F_{(10,60)}$	= 13.46	< .001	*	.079	*
Distance:Weight	$F_{(10,60)}$	= 20.39	< .001	*	.1	*
Mode:Duration	$F_{(1,6)}$	= 1.63	.25		.001	
Observations:Duration	$F_{(2,12)}$	= 10.48	.002	*	.025	
Distance:Duration	$F_{(2,12)}$	= 0.65	.54		.002	
Weight:Duration	$F_{(5,30)}$	= 4.67	.003	*	.015	
Mode:Observations:Distance	$F_{(4,24)}$	= 5.32	.003	*	.008	
Mode:Observations:Weight	$F_{(10,60)}$	= 2.62	.01	*	.013	
Mode:Distance:Weight	$F_{(10,60)}$	= 22.30	< .001	*	.077	*
Observations:Distance:Weight	$F_{(20,120)}$	= 2.49	.001	*	.018	
Mode:Observations:Duration	$F_{(2,12)}$	= 0.16	.85		.0003	
Mode:Distance:Duration	$F_{(2,12)}$	= 24.06	< .001	*	.01	
Observations:Distance:Duration	$F_{(4,24)}$	= 2.67	.057		.009	
Mode:Weight:Duration	$F_{(5,30)}$	= 6.69	< .001	*	.022	
Observations:Weight:Duration	$F_{(10,60)}$	= 1.49	.17		.008	
Distance:Weight:Duration	$F_{(10,60)}$	= 3.00	.004	*	.008	
Mode:Observations:Distance:Weight	$F_{(20,120)}$	= 4.76	< .001	*	.026	
Mode:Observations:Distance:Duration	$F_{(4,24)}$	= 0.90	.48		.002	
Mode:Observations:Weight:Duration	$F_{(10,60)}$	= 0.61	.8		.004	
Mode:Distance:Weight:Duration	$F_{(10,60)}$	= 4.19	< .001	*	.009	
Observations:Distance:Weight:Duration	$F_{(20,120)}$	= 2.34	.002	*	.013	
Mode:Observations:Distance:Weight:Duration	$F_{(20,120)}$	= 1.54	.081		.008	

Significance of effects of CSSM configuration factors on Accuracy, using 216 CMf / CPf configurations. (2 modes, 3 observations, 3 distances, 6 weights, 2 durations.)
