# Supplementary material for: Computational State Space Models for Activity and Intention Recognition. A Feasibility Study
Source: PLoS One. 2014 Nov 5;9(11):e109381. doi: 10.1371/journal.pone.0109381 (PMC4220990; doi:10.1371/journal.pone.0109381)
Supplement: Appendix S1 — Notational conventions and abbreviations. (PDF) [file pone.0109381.s023.pdf]

S1 Notational conventions and abbreviations

For random variables X, Y with value domains $\mathcal{X}$, $\mathcal{Y}$ and conditional density $p(X \mid Y)$ we abbreviate $p(X{=}x \mid Y{=}y)$ as $p(x \mid y)$ with realizations $x \in \mathcal{X}, y \in \mathcal{Y}$ in case the lower case realization names unambiguously determine the corresponding random variables. For some distributions explicit names are chosen such that, for instance, $\varphi(x \mid y) := p(x \mid y)$ implies that $\varphi(a, b) = p(X{=}a, Y{=}b)$. Somewhat careless we say "the distribution $p(X)$" when $p(X)$ is really a density function.

A boolean expression has either the value 1 (logical true) or 0 (false). The Iverson bracket $[\alpha]$ has the value 1 in case the logical statement α is true, otherwise 0. The vector $(a_1, \ldots, a_n)$ is abbreviated as $a_{1:n}$.

Sometimes, an Iverson bracket $[c = x]$ is used as distribution, where c is some constant and $x \in \mathcal{X}$ ranges over a set of values. If $\mathcal{X}$ is a finite set, $[c = x]$ is simply a probability mass function. If $\mathcal{X}$ is continuous, $[c = x]$ represents a δ distribution placed at c.

The notation $(CI_{.95} = x, y)$ is used for reporting the confidence interval $[x, y]$ at the .05 α level. Wilcoxon signed rank test is used for testing significant difference of accuracy between models or varying parameterizations, where $(V_{(n)},\ p < x)$ denotes the statistics and p-value for the Wilcoxon test with n pairs.

Table 1. Abbreviations and notations used in the article.

Abbreviation	Meaning
ANOVA	analysis of variance
aLTS	annotation LTS
$CI_{.95}$	95% confidence interval
CSSM	computational state space model
DBN	dynamic Bayesian network
DTW	dynamic time warping
HMM	hidden Markov model
$F_{(a,b)}$	F statistic with a and b degrees of freedom
i.i.d	independent and identically distributed
iLTS	inference LTS
IMU	inertial measurement unit
IQR	interquartile range
JSD	Jensen-Shannon distance
LTS	labeled transition system
MAP	maximum a-posteriori
MF	marginal filter
PF	particle filter
QDA	quadratic discriminant analysis
rANOVA	repeated measures ANOVA
SD	standard deviation
SSM	state space model
$t_{(d)}$	t statistic with d degrees of freedom
$V_{(n)}$	Wilcoxon signed rank statistic with n pairs
