# Supplementary material for: Computational State Space Models for Activity and Intention Recognition. A Feasibility Study
Source: PLoS One. 2014 Nov 5;9(11):e109381. doi: 10.1371/journal.pone.0109381 (PMC4220990; doi:10.1371/journal.pone.0109381)
Supplement: Appendix S2 — Computational state space models. (PDF) [file pone.0109381.s024.pdf]

S2 Computational state space models

This section provides a brief review of the pertinent notions of CSSMs from the perspective of intention recognition (IR) and activity recognition (AR). We draw on concepts of action languages [1], symbolic planning [2, p. 366], and plan recognition [3]. Our discussion of IR and AR is focused on the objective of introducing the probabilistic machinery of CSSMs; for a more general perspective on the field of IR we refer the reader to available surveys [4–7].

S2.1 Actions, states, intentions, plans

We consider dynamic systems whose behavior can be formally captured by the notion of labeled transition systems (LTS). An LTS is a triple $(\mathcal{S}, \mathcal{A}, \rightarrow)$ where $\mathcal{S}$ is a set of states, $\mathcal{A}$ a set of labels (which we in this context usually call "actions") and $\rightarrow \subseteq \mathcal{S} \times \mathcal{A} \times \mathcal{S}$ a ternary relation, representing the labeled transitions. If for two states $s, s' \in \mathcal{S}$ and an action $a \in \mathcal{A}$ we have that $(s, a, s') \in \rightarrow$, we say that s' is *reachable* from s by a, or s' is the result of applying a in s. This is written as $s \stackrel{a}{\rightarrow} s'$. If for a given $s \in \mathcal{S}$ and $a \in \mathcal{A}$ there exists an $s' \in \mathcal{S}$ such that $s \stackrel{a}{\rightarrow} s'$, we say that a is *applicable* to s, or that s fulfills the precondition of a. If for any pair (s, a) there exists at most one s' such that $s \stackrel{a}{\rightarrow} s'$, we say that a is *deterministic*, resp. that a has deterministic effects. In this case we can consider a to be a function defined by $s' = a(s) \iff s \stackrel{a}{\rightarrow} s'$. If all actions are deterministic, the LTS itself is deterministic. For simplicity, we consider only LTS with deterministic effects (we will introduce nondeterminism later via the nondeterministic choice between the different actions that may be applicable to a state). Note that $\mathcal{S}$ and $\mathcal{A}$ need not be finite.

A *goal* is a set of states. A *plan* $l = (a_1, \ldots, a_n)$ for a goal $g \subseteq \mathcal{S}$ and an initial state $s_0 \in \mathcal{S}$ is a finite sequence of actions a_i such that $s_n \in g$ where $s_n = a_n(\cdots a_2(a_1(s_0))\cdots)$. We say that l *achieves* g. Furthermore, we say that an agent executing a plan l that achieves a goal g has the *intention* to achieve g. Using this notion of intention we can say that intentions are isomorphic to goals. We can recognize an agent's intentions by observing the agent to work on a plan known to achieve some goal g (*intention recognition*). Informally, we refer to the number of alternative paths (between two states) as (local) "dispersion" of an LTS.

S2.2 Action languages and latently infinite LTS

It is easy to arrive at LTS where $\mathcal{S}$ and $\rightarrow$ are infinite, even though $\mathcal{A}$ is finite – for instance by introducing states that represent counter values and actions that increment such counters (the natural numbers and the successor function are such an LTS). In the domain of intelligent assistive systems, protagonist activities such as setting a table by incrementally moving items from the kitchen to the dining room result in this counting behavior. Such LTS can not be represented by explicit enumeration of states and transitions. However, in case every action can be represented by an *algorithm*, defined in a suitable algorithmic language (which we call "computational action language"), then also these LTS have a finite representation. An action here can be considered a function that computes (generates) a resulting state from its origin state: the LTS graph is incrementally expanded during computation. As long as only a finite subset of states needs to be considered in a given intention recognition task, computations on such latently infinite systems remain feasible. Clearly, a computational action language – possibly Turing complete – is also helpful for providing a compact representation of a finite, yet bulky LTS.

Informally, a basic model for an action language can be defined as follow:

- There is a set of *variable names* and a set of *value domains* whose disjoint union describes the set of possible values.
- A state is a finite map of variable names to values. In unstructured, atomic state models, as used in HMMs, state values are opaque labels that only support to compare for identity. In order to

allow computations on states (such as generating a new state from a given state), state values have to provide a suitable algebraic structure, such as given by this slot-value model.

In assistive settings, structured states enable a straightforward mechanism for guidance in case of erroneous, unexpected, or ineffective behavior: planning mechanisms can readily be used for computing a route from the current state to the original goal state. This option is not available in unstructured state models. Here, contingency plans have to be enumerated explicitly.

- We assume that the language allows to write expressions that may refer to state variables. Let E be the set of all expressions. We write $e(s)$ to denote the value that an expression $e \in E$ takes on in the context of a state $s \in S$ (that is: the value of e where the values of variables in e are taken from the state s).
- An action a is defined by
    1. The precondition π_a: a boolean expression on state variables. If the value $\pi_a(s)$ for a given state s is true, then we say that a is applicable to s. We write this as $s \models \pi_a$.
    2. The effect ϵ_a: a finite map of variable names v_i to expressions e_i. If a is applied to a state s, a new state s' results from s by assigning the value of $e_i(s)$ to each v_i and by copying the remaining mapping from s.

The STRIPS language [8] is a particularly simple instance of an action language (although not Turing complete), where add and delete lists of STRIPS actions effectively model the assignment of "true" or "false" to variables. PDDL 2.1 [9] is another example from the planning domain, supporting numerical expressions and infinite value domains of variables. Other examples are rule-based expert system languages such as CLIPS [10] or the production rule language of the ACT-R cognitive architecture [11], both Turing complete.

Action languages such as PDDL typically support *action schemata* – action templates that contain template variables from which actions can be constructed by instantiating these template variables with domain values. A single schema represents the set of actions that can be created by instantiating its variables. To differentiate actions – which have no template parameters – from action schemata, the former are sometimes called "ground actions". The unique label of a ground action a created from a schema is the tuple consisting of schema name and the parameter values used for instantiation.

S2.3 CSSMs: Probabilistic interpretation

State space models (SSMs) [12] are a general class of probabilistic models where a sequence of observations is explained by the sequential evolution of a hidden state variable. Let $\mathcal{X}$ be some set of states, let $X_{1:t}$ be a sequence of random variables with value domain $\mathcal{X}$. Furthermore, let $\mathcal{Y}$ be a set of observations and $Y_{1:t}$ a sequence of random variables with value domain $\mathcal{Y}$. Then the joint distribution $p(x_{1:t}, y_{1:t})$ can be described by an SSM if it recursively (over time t) factorizes into a *transition model* $p(x_t \mid x_{t-1})$ and an *observation model* $p(y_t \mid x_t)$, that is $p(x_{1:t}, y_{1:t}) = p(y_1 \mid x_1)\, p(x_1) \prod_{i=2}^{t} \big(p(y_t \mid x_t)\, p(x_t \mid x_{t-1})\big)$. The SSM is stationary if $p(x_t \mid x_{t-1}) = p(x' \mid x)$ and $p(y_t \mid x_t) = p(y \mid x)$ for random variables X', X with value domain $\mathcal{X}$ and Y with value domain $\mathcal{Y}$. Hidden Markov models, Kalman filters, and dynamic Bayesian networks are instances of SSMs that use specific function families for representing the transition model and the observation model. Given a sequence of observations $y_{1:t}$ with $y_i \in \mathcal{Y}$, we can employ the combination of prediction (computing the next state based on past observations) and correction (computing the current state based on the current observation and the prediction) for recursive state estimation, as given by the two equations

$$p(x_t \mid y_{1:t-1}) = \int_{x_{t-1} \in \mathcal{X}} p(x_t \mid x_{t-1})\, p(x_{t-1} \mid y_{1:t-1}) dx_{t-1} \tag{1}$$

$$p(x_t \mid y_{1:t}) = \frac{p(y_t \mid x_t)\, p(x_t \mid y_{1:t-1})}{p(y_t \mid y_{1:t-1})} \tag{2}$$

where $p(x_t \mid y_{1:t})$ is known as *marginal filtering distribution* (correspondingly, $p(x_{1:t} \mid y_{1:t})$ is the joint filtering distribution, as it jointly estimates the whole state series $x_{1:t}$). The underlying idea of *computational* state space models (CSSMs) is to use computational action languages for representing the transition distribution. This approach is interesting when the process under observation can be considered as performing some kind of sequential "computation", including such phenomena as goal directed behavior of human protagonists.

A CSSM is defined by an LTS $(\mathcal{S}, \mathcal{A}, \rightarrow)$ with a set of goals $\mathcal{G}$ as follows. One defines $\mathcal{X} := \mathcal{S} \times \mathcal{A} \times \mathcal{G}$ and $X := (S, A, G)$. The transition model becomes:

$$\begin{aligned} p(x' \mid x) &= p(s', a', g' \mid x) \\ &= p(s' \mid a', g', x)\, p(a' \mid g', x)\, p(g' \mid x) \\ &= p(s' \mid a', s)\, p(a' \mid g', s, a)\, p(g' \mid x), \end{aligned}$$

where the last step reflects two simplifying design assumptions: (i) The LTS action a' selected for generating the new LTS state s' from the previous state s does not depend on the previous goal g. (ii) The new LTS state s' depends only on the previous LTS state s and the action a' applied to this state. Specifically, if the underlying LTS is deterministic, then $p(s' \mid a', s) = [s' = a'(s)]$. (These assumptions are not strictly necessary, but they simplify the resulting model structure.) We call $\gamma(a' \mid g', s, a) := p(a' \mid g', s, a)$ the action selection distribution. If the underlying LTS is deterministic and if goals are fixed, then all nondeterminism is solely introduced by the action selection distribution, which models the protagonist's freedom of choice.

As the probabilistic state variable X contains both the LTS state S and the LTS action A, an observation model $p(y \mid x) \equiv p(y \mid s, a, g)$ may be built on observations of LTS states, of LTS actions, or combinations of both. One often has the assumption that the observations factor into independent state and action observations $Y = (W, Z)$, so that state observations W only depend on the state S and action observations Z only depend on the action A. This leads to the additional simplifying independence $p(y \mid x) \equiv p(w, z \mid s, a, g) = p(w \mid s)\, p(z \mid a)$. This has been used for example by [13]. The availability of state observations for inference is an interesting aspect of CSSMs, as it is no longer necessary to assume that protagonist actions can be directly observed. At the same time, state estimation via the filtering equation allows to make statements about S even in case only action observations are available.

S2.4 Action selection

The action selection distribution γ models the non-deterministic behavior of protagonists in the case that multiple actions are applicable to a given situation. Specifically for human protagonists, this behavior is potentially influenced by a large set of factors [14] that are current target of research on human behavior. They encompass decision theoretic quantities, such as an "action's utility" in reaching the goal from the given state (being used, for instance, in the ACT-R cognitive architecture [11]), as well as situation-based conflict resolution strategies from the domain of rule-based expert systems, such as "specificity" [15].

Independent of the specific number and nature of the different factors, they can be combined using a log-linear model [16], so that the action selection distribution γ is given by

$$\gamma(a' \mid g', s, a) \propto \exp\left(\sum_{i \in \mathcal{I}} \lambda_i f_i(a', g', s, a) \right) \tag{3}$$

where the functions $f_i : \mathcal{A} \times \mathcal{G} \times S \times \mathcal{A} \rightarrow \mathbb{R}$ represent the different factors and the $\lambda_i \in \mathbb{R}$ their respective weights, for some index set $\mathcal{I}$. For instance, let $\delta_{\mathcal{A}}(s, g)$ be the distance (or, more general, the cost of the optimal path) from state s to goal g given the action set $\mathcal{A}$ and $w(s, a', s')$ the length (cost) associated with the LTS edge $s \xrightarrow{a'} s'$. Define $Q_{g'}(a', s) := w(s, a', a'(s)) + \delta(a'(s), g')$. Then the action selection model given by [17], where $\gamma(a' \mid g', s, a) \propto \exp\left(\beta\, Q_{g'}(a', s)\right)$ (adapted from eq. (8) in [17]) naturally arises as

special case of (3) by setting $\mathcal{I} = \{\delta\}$ and $f_\delta(a', g', s, a) := Q_{g'}(a', s)$ as well as $\lambda_\delta := \beta$. Likewise, we can incorporate concepts such as "specificity", a function $\sigma : \mathcal{A} \times \mathcal{S} \to \mathbb{R}$, where $\sigma(a', s)$ quantifies how specific the precondition of a' is for s, as another factor of (3). Restricting action selection to only those actions that are applicable to s, where $s \models \pi_{a'}$, can be seen as very simple special case of specificity. One observes that $[s \models \pi_{a'}] = \exp\big(f_\pi(a', g', s, a)\big)$ where

$$f_\pi(a', g', s, a) := \begin{cases} 0, & \text{if } s \models \pi_{a'} \\ -\infty, & \text{otherwise.} \end{cases}$$

So this restriction can easily be embedded as another feature into (3), with weight $\lambda_\pi := 1$.

We will therefore assume that (3) represents the basic functional structure of the action selection distribution in CSSMs. In the face of a large set of factors not yet fully understood, the interesting aspect of using the log-linear model is that it allows (i) factors f_i that interact in arbitrary ways, and (ii) the estimation of the factor weights λ_i from training data. Thus, for the purpose of using CSSMs, it is not necessary to wait for research to arrive at a final set of independent factors governing human action selection.

References

1. Gelfond M, Lifschitz V (1998) Action languages. Linköping Electronic Articles in Computer and Information Science 3.

2. Russell S, Norvig P (2010) Artificial Intelligence – A Modern Approach. Prentice Hall, third edition.

3. Kautz HA (1991) A formal theory of plan recognition and its implementation. In: Brachman RJ, Allen JF, Kautz HA, Pelavin RN, Tenenberg JD, editors, Reasoning about Plans, San Francisco, CA, USA: Morgan Kaufmann Publishers Inc. pp. 69–126.

4. Aggarwal JK, Ryoo MS (2011) Human activity analysis: A review. ACM Computing Surveys 43: 16:1–16:43.

5. Chen L, Hoey J, Nugent C, Cook D, Yu Z (2012) Sensor-based activity recognition. IEEE Transactions on Systems, Man, and Cybernetics, Part C: Applications and Reviews 42: 790–808.

6. Han TA, Pereira LM (2013) State-of-the-art of intention recognition and its use in decision making. AI Communications 26: 237–246.

7. Sadri F (2011) Logic-based approaches to intention recognition. In: Chong NY, Mastrogiovanni F, editors, Handbook of Research on Ambient Intelligence: Trends and Perspectives, IGI Global. pp. 346–375.

8. Fikes RE, Nilsson NJ (1971) Strips: A new approach to the application of theorem proving to problem solving. In: Proceedings of the second International Joint Conference on Artificial Intelligence (IJCAI). San Francisco, CA, USA: Morgan Kaufmann Publishers Inc., pp. 608–620.

9. Fox M, Long D (2003) PDDL2.1: An extension to PDDL for expressing temporal planning domains. Journal of Artificial Intelligence Research (JAIR) 20: 61–124.

10. Giarratano JC, Riley GD (2004) Expert Systems: Principles and Programming. Boston, MA: Course Technology, fourth edition.

11. Anderson JR, Bothell D, Byrne MD, Douglass S, Lebiere C, et al. (2004) An integrated theory of the mind. Psychological Review 111: 1036–1060.

12. Koller D, Friedman N (2009) Probabilistic Graphical Models. Cambridge, MA: MIT Press.

13. Hoey J, Plötz T, Jackson D, Monk A, Pham C, et al. (2011) Rapid specification and automated generation of prompting systems to assist people with dementia. Pervasive and Mobile Computing 7: 299–318.

14. Prescott TJ, Bryson JJ, Seth AK (2007) Theme issue 'modelling natural action selection'. Philosphical Transactions of the Royal Society B – Biological Sciences 362: 1521-1721.

15. Krüger F, Yordanova K, Burghardt C, Kirste T (2012) Towards creating assistive software by employing human behavior models. Journal of Ambient Intelligence and Smart Environments 4: 209–226.

16. Berger A, Pietra SD, Pietra VD (1996) A maximum entropy approach to natural language processing. Computational Linguistics 22: 40–71.

17. Ramírez M, Geffner H (2011) Goal recognition over POMDPs: Inferring the intention of a POMDP agent. In: Proceedings of the 22nd International Joint Conference on Artificial Intelligence (IJCAI). Barcelona, Spain, pp. 2009-2014.
