# Supplementary material for: Computational State Space Models for Activity and Intention Recognition. A Feasibility Study
Source: PLoS One. 2014 Nov 5;9(11):e109381. doi: 10.1371/journal.pone.0109381 (PMC4220990; doi:10.1371/journal.pone.0109381)
Supplement: Appendix S3 — Annotation procedure and annotation ontology. (PDF) [file pone.0109381.s025.pdf]

S3 Annotation procedure and annotation ontology

S3.1 Underlying rationale.

Objective of annotation is to provide a target label ℓ_t for every observation y_t such that methods for supervised learning can be applied. In addition, comparing target values with the values estimated from observation data is used for quantifying the performance of the estimation procedure. These labels are called "ground truth", as they conceptually provide a symbolic representation of the true state of the world at time t. However, in reality, labels are a finite set $\mathcal{L} := \{\ell_1, \ldots, \ell_n\}$ where besides the equality relation no other algebraic structure on $\mathcal{L}$ exists. In current annotation practice, each label value $\ell \in \mathcal{L}$ is accompanied by some textual description (such as "take knife"), that supports the annotator in selecting what label ℓ_t to attach to an observation y_t. This description looks like a symbolic representation – but this often is an illusion: There is no formal set of constraints on the structure of label sequences that would enforce them to reflect constraints on causality implied by the labels' textual descriptions. The language of formally valid label sequences is $\mathcal{L}^*$ itself.

There is nothing that denies an annotator to produce the label sequence "go from A to B","wait","go from C to D". Under the natural interpretation of these textual labels, such annotations represent acausal behavior. Instances of this phenomenon can readily be found in the data provided by [1–3]. As long as models do not aim at exploiting the causal structure of behavior, such annotation oddities are no problem. However, in order to use CSSMs, we must rely on "ground truth" to indeed represent a true causal sequence of states and actions. This therefore requires that already the annotation is based on a formal model of causal behavior. Causality not available in the annotation model can not be reliably exploited in an inference model. The consequence of this is that already the annotations must be based on an LTS, the *annotation LTS* (aLTS). A second consequence is that the algebraic refinement relations that hold between the aLTS and the CSSM LTS (the *inference* LTS, iLTS) determine the CSSM's capability to correctly estimate annotation sequences – for instance, unless the iLTS is a refinement of the aLTS, the CSSM model will not be able to differentiate between certain states and actions that are discernible in the aLTS. Thus, from a certain viewpoint, CSSM model development already begins at the annotation stage.

In addition to these considerations, annotations should be agnostic to the capabilities of the underlying sensors. Some researchers select the annotation dictionary based on the expected capability of the sensor used in the experiment to identify such actions or states [4–6]. For instance, temperature sensors in the shower, pressure sensors in the bed, and reed switches in doors strongly correlate with annotated activities showering, sleeping, and opening / closing. This approach may seem convenient at first glance, but was rejected for two reasons: (i) it exaggerates the resulting model's discriminative capabilities (as "difficult" things are simply dropped from the target set), and (ii) it is in fundamental disagreement with the "philosophy" of CSSMs, as the resulting annotation dictionary – and the data set annotations – is not reusable for other sensor modalities.

S3.2 aLTS development method and data set annotation.

For aLTS model development, a simplified process of model driven engineering [7] was used to combine information from the empirical samples with the background knowledge of the domain expert.

1. From the video log the atomic domain objects and their state variables were identified by the domain expert. All physical entities were considered as domain objects that were independently manipulated by the protagonist (including the protagonist). Roughly, objects x, y are independent, if a specific manipulation of x does not imply another specific manipulation of y and vice versa. Although this is not an exact definition it was sufficient for the purpose of this study.
   A deliberate design choice was to avoid aLTS state variables with continuous domains, as this would introduce another source of variance into the model that would further increase the difficulty of

identifying the different causes of model performance. Therefore, protagonists would be hungry or ¬hungry, protagonist locations would be elements of some (small) finite set, glasses would be filled or ¬filled, etc.

2. Atomic actions were identified from the resulting state sequences. Atomic actions are those actions that do not contain other actions that have an effect on the state of domain objects. Thus, the notion of atomicity is essentially implied by the granularity of the aLTS state model. Actions were identified from the video log by assigning an action to every frame sequence that would delimit a state change to an aLTS object.
3. aLTS actions were abstracted into action schemata by (a) identifying classes of domain objects and (b) classes of domain actions such that all elements of an aLTS action class could be generated from the corresponding action schema by instantiating the parameters of this action schema with elements of suitable domain object classes. These action schemata would represent the causality of the domain using a precondition–effect model.

   For the purpose of this study, natural language was chosen as source of intuitive prior knowledge, by allowing the aLTS designer to select a small set of verbs (such as take) as labels for the identified action schemata. Following, these aLTS action schema labels are referred to as *action classes*.

   Typically, in this abstraction process a set of state transitions such as $\{(x_{t-1}{=}1 \mapsto x_t{:=}1), (x_{t-1}{=}2 \mapsto x_t{:=}4), (x_{t-1}{=}3 \mapsto x_t{:=}9)\}$ would be represented by a single action such as $x_t := x_{t-1}^2$, replacing explicit values with a *computation* that generates the desired values as needed. These computational representations are a powerful mechanism for generalization, that extends the scope of the model towards an infinite range of states. Inventing computational actions requires inductive reasoning; it adds knowledge not deducible from the training data. Usually, this will be prior knowledge available to the aLTS designer.
4. Finally, the annotation sequences defined for the data sets were checked against the aLTS actions to ensure that each annotation sequence would be a valid path through the LTS defined by the aLTS actions.

S3.3 Complex interleaving.

Although many actions could be considered as deterministic, non-deterministic effects were used to resolve some non-trivial interleaving patterns. For instance, consider the interleaved process of eating and drinking. In case no other actions are allowed to intervene, this can be summarized by the following regular grammar:

$$((\mathsf{take(spoon,table)}, \mathsf{eat}, \mathsf{put(spoon,table)}) \mid (\mathsf{take(glass,table)}, \mathsf{drink}, \mathsf{put(glass,table)}))^*$$

Clearly, the last eat (resp. drink) will achieve ¬hungry (resp. ¬thirsty). To model this behavior, the ¬hungry effect of eat was considered to be a probabilistic effect, one of the preconditions of eat being hungry. Note that the transition from eat to drink and vice versa requires certain intervening actions (e.g., put(spoon,table), take(glass,table)). The interleaving of "eating" and "drinking" therefore can not be handled by a simple interleaved execution of two parallel actions eat and drink (as proposed – for a different scenario – in [8]). For the same reason, the duration of eat can not be represented by a single durative PDDL action.

Implementationally, a single action a with precondition π_a and a finite distribution over n probabilistic effects $\epsilon_a^{(i)}$ having probability p_i was approximated by a set of n actions a_i with single preconditions $\pi_{a_i} = \pi_a$ and deterministic effects $\epsilon_{a_i} := \epsilon_a^{(i)}$, where the relative probability of selecting a_i depends on p_i.

References

1. Regneri M, Rohrbach M, Wetzel D, Thater S, Schiele B, et al. (2013) Grounding action descriptions in videos. Transactions of the Association for Computational Linguistics (TACL) 1: 25–36.

2. Spriggs E, de La Torre F, Hebert M (2009) Temporal segmentation and activity classification from first-person sensing. In: IEEE Workshop on Egocentric Vision, in conjunction with CVPR 2009. Miami, Florida, USA, pp. 17–24.

3. de la Torre F, Hodgins J, Montano J, Valcarcel S, Forcada R, et al. (2009) Guide to the carnegie mellon university multimodal activity (CMU-MMAC) database. Technical Report CMU-RI-TR-08-22, Robotics Institute, Carnegie Mellon University.

4. Donnelly M, Magherini T, Nugent C, Cruciani F, Paggetti C (2011) Annotating sensor data to identify activities of daily living. In: Abdulrazak B, Giroux S, Bouchard B, Pigot H, Mokhtari M, editors, Toward Useful Services for Elderly and People with Disabilities, Springer Berlin Heidelberg, volume 6719 of *Lecture Notes in Computer Science*. pp. 41–48.

5. van Kasteren TLM, Kröse BJA (2009) A sensing and annotation system for recording datasets in multiple homes. In: Proceedings of the 27th Annual Conference on Human Factors and Computing Systems. Boston, USA, pp. 4763–4766.

6. Stikic M, Van Laerhoven K (2007) Recording housekeeping activities with situated tags and wrist-worn sensors: Experiment setup and issues encountered. In: Proceedings of the first International Workshop on Wireless Sensor Networks for Health Care (WSNHC). Braunschweig, Germany.

7. Fondement F, Silaghi R (2004) Defining model driven engineering processes. In: Proceedings of the third Workshop in Software Model Engineering Satellite workshop at the seventh International Conference on the UML. Lisabon, Portugal.

8. Shi Y, Huang Y, Minnen D, Bobick A, Essa I (2004) Propagation networks for recognition of partially ordered sequential action. In: Proceedings of the IEEE Computer Society Conference on Computer Vision and Pattern Recognition (CVPR). Washington DC, USA, pp. 862–869.
