# Supplementary material for: Computational State Space Models for Activity and Intention Recognition. A Feasibility Study
Source: PLoS One. 2014 Nov 5;9(11):e109381. doi: 10.1371/journal.pone.0109381 (PMC4220990; doi:10.1371/journal.pone.0109381)
Supplement: Appendix S4 — Detailed model description. (PDF) [file pone.0109381.s026.pdf]

S4 Detailed model description

S4.1 Statistical model

The statistical model formally describes the framework and assumptions of the model for the kitchen task scenario. First, an extension of the CSSM to correctly handle durative actions is described. Then the overall DBN interpretation of the model is given. Afterward, the sensor model, duration model, and action selection heuristics are discussed in detail.

S4.1.1 Durative actions

The model given in Appendix S2 assumes that actions take a single time step, where progression of time is given by observation events. This requires that sensor data – usually some kind of real time signal – is already partitioned into segments that correspond to individual actions, each segment representing a single observation. In this case, the subscript t in y_t simply counts the number of actions that have been observed. However, in general correct segmentation needs additional context information for disambiguation [1], such as the possible sequence of actions. The assumption that the CSSM will be presented with observations that represent complete action sightings, as for instance used in all but one of the previous CSSM studies (Sec. 1.3.2), thus hides important aspects of the real-world inference problem.

Therefore, a model was chosen where multiple observations may correspond to a single action. This model introduces real-valued random variables U and V that represent the starting time of an action A and its duration. The action duration density $\tau(v \mid a, u) := p(V \mid A, U)$ defines the probability that action A stops at time V if started at time U. Let $F_\tau(v \mid a, u)$ be the cumulative distribution function of τ. The probability that an action's duration lies in the interval $(v, v']$ then is $\zeta_{a,u}^{v,v'} := P(v < V \leq v' \mid a, u) = F_\tau(v' \mid a, u) - F_\tau(v \mid a, u)$. If this duration is known to be greater than v, the corresponding conditional probability is $P(v < V \leq v' \mid a, u, V > v) = \zeta_{a,u}^{v,v'} / \zeta_{a,u}^{v,\infty}$.

Note that durative actions will increase state space complexity.

S4.1.2 Overall CSSM model structure

For the probabilistic model of Sec. 1.2 (see Appendix S2 for details), a DBN with the structure given in Fig. 1 was used. $Y_t = (W_t, Z_t)$ is the observation data for time step t. V_t is the associated time stamp, required to be strictly increasing. As discussed in Appendix S2, state and action observations were assumed to be conditionally independent, i. e., $p(y_t \mid a_t, s_t) = p(w_t \mid s_t)\, p(z_t \mid a_t)$.

$X_t = (A_t, D_t, G_t, S_t, U_t)$ defines the hidden state. For this study, G_t, the current goal, could be assumed to be constant, so that $p(g_t \mid x_{t-1}) = [g_t = g_{t-1}]$. This allowed to efficiently precompute goal distance values, which depend on G_t. The boolean flag D_t signals termination of A_{t-1} in the interval $(v_{t-1}, v_t]$. It is a Bernoulli random variable defined by $p(D_t{=}1 \mid v_t, v_{t-1}, a_{t-1}, u_{t-1}) = \zeta_t / \zeta_{a_{t-1},u_{t-1}}^{v_{t-1},\infty}$ where $\zeta_t := \zeta_{a_{t-1},u_{t-1}}^{v_{t-1},v_t}$. The variable D_t introduces a context-specific independence [2] into the DBN: if $d_t = 0$, then A_t, S_t, and U_t carry over their values from the previous state and are independent of their other parents. Otherwise, new values for A_t, S_t, and U_t are selected as follows:

A new action is selected according to γ by: $p(a_t \mid d_t = 1, s_{t-1}, a_{t-1}, g_t) = \gamma(a_t \mid g_t, s_{t-1}, a_{t-1})$. U_t is the starting time of action A_t, given by $p(u_t \mid d_t = 1, u_{t-1}, v_t, v_{t-1}, a_{t-1}) = \bar{\tau}(u_t \mid u_{t-1}, v_t, v_{t-1}, a_{t-1})$, where $\bar{\tau}(u_t \mid u_{t-1}, v_t, v_{t-1}, a_{t-1}) := [v_{t-1} < u_t \leq v_t]\, \tau(u_t \mid a_{t-1}, u_{t-1}) / \zeta_t$ is the truncated duration density, constrained by $v_{t-1} < u_t \leq v_t$. S_t is the LTS state for time step t: either the result of applying the new action to the previous state, or by carrying over the old state. For the purpose of this study, actions could be assumed to be deterministic and with instantaneous effect, giving the simple model $p(s_t \mid d_t = 1, s_{t-1}, a_t) = [s_t = a_t(s_{t-1})]$. As outlined for aLTS model development in Appendix S3, non-deterministic effects for modeling complex interleaving were represented by multiple actions.

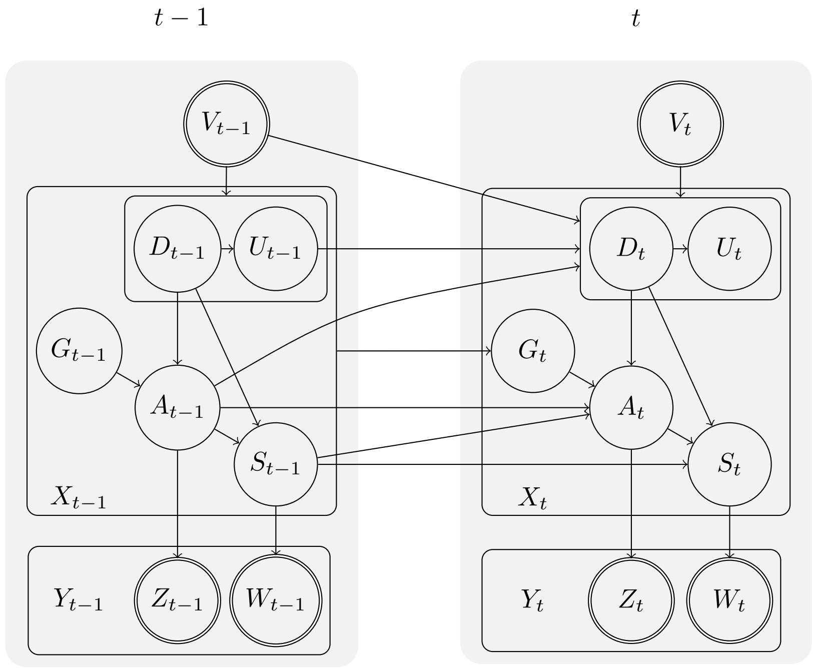

Figure 1. CSSM DBN structure. Boxes represent tuples of random variables. An arc starting / ending at a box (= a tuple) represents a set of arcs connected to the tuple's components. Nodes with double outline signify observed random variables.

S4.1.3 Sensor models and scrambling

As construction of an elaborate observation model was not in the focus of this study, the following model was selected for its conceptual and computational simplicity: all actions a of a given class $c = class(a)$ share the same observation distribution, such that $p(z \mid a) := p(z \mid c)$ and the distributions $p(z \mid c) := N(z \mid \mu_c, \Sigma_c)$ were represented by multivariate normal distributions with unconstrained covariance Σ_c. The parameters μ_c, Σ_c were computed from the pool of all observations annotated with class c from all data sets. Although there is no reason to believe that the observation data is particularly well represented by this model, it was found to perform reasonably well in the baseline models, justifying its further use in this study. The 16 action classes can be seen in Fig. 6 or Fig. S3. For example, an action class is TAKE, while the actions belonging to it are take carrot, take bottle, take spoon etc.

A potential significant limitation of this model is its stationarity with respect to durative actions, which might be violated by the sensor data. A "run" for class c is a consecutive sequence of observations labeled with c. A run (often) represents an action that lasts several time steps. It might be the case that observations in the middle of a run are more "typical" for action class c than at the beginning (or end) of the run, where the transition to a different action class takes place and both kinds of motions are mixed. In this case, the expected probability of observations should increase from the run borders towards the run center. This effect should be specifically prominent for actions with long durations (which have long stable "center periods"). This could, for instance, impact the ability to correctly detect the start of such actions and eventually degrade recognition performance.

Conceptually, a better option to solve this problem is either to include temporal information into the observations [3] or – more general – to use a hierarchical approach, where the temporal structure of an action's observations are represented by suitable sub-models [4]. A much simpler potential solution is

to scramble observations within a run. This will destroy order effects and other dependencies between observations in a run. Clearly this approach is only possible in offline situations with data where the runs are already delimited (either by annotations or by some suitable segmentation algorithm). However, regarding the study objectives, the use of scrambling is considered a justified surrogate for defining appropriate sub-models.

As alternative to the IMU sensors, which give continuous-domain observations of the A state component, a location-based model was set up, giving categorical observations (place names) of the S state component. Motivation for introducing this alternative observation model was to demonstrate that CSSMs allow an exchange of observed X component without requiring adaptation of the system model. In addition, it was of interest to:

(i) Test in how far a switch from continuous (IMU) to discrete (place names) observation modalities introduces additional challenges for inference.

(ii) Provide a non-peaked observation model that assigns a non-negligible probability to every state, in order to test susceptibility of inference method to this observation model property.

With respect to these objectives, a very simple model was considered sufficient, where the locations of the protagonist (3 places) and the food (6 places) were used (see Tbl. S5). For the conditional probability $p(z_t \mid s_t)$ the value 10^{-6} was used in case the locations observations z_t did not match the locations found in s_t and essentially 1 otherwise. The observations themselves were computed from the aLTS model by stepwise execution of the aLTS ground actions recorded in the annotations and using the location slot values of the observed objects in the resulting state. While being realistic with respect to the temporal and causal structure of the underlying human activity, these synthetic observations are unrealistic concerning the precision of temporal alignment and error model; they can be expected to exaggerate the achievable precision. Nevertheless, as this was a convenient mechanism to obtain data for new observation models for simple comparison purposes, its use was considered legitimate.

S4.1.4 Duration model

For simplification it was assumed that all actions of a given class share the same action duration distribution and that the duration of an action does not depend on the time the action has started. Therefore, $\tau(v \mid a, u) = \tau^*(v - u \mid class(a))$ where $\tau^*(d \mid c)$ is the class-specific duration distribution. As there is yet no prior knowledge on action durations, it was necessary to use the durations found in the training data as proxy.

Note that duration distributions $\tau^*(d \mid c)$ with large or even infinite support increase the branching factor. In order to determine this effect on inference performance, an instance based model (with finite support) and a parametric duration model (infinite support) was built. Let $(d_{c,1}, \ldots, d_{c,n_c})$ be the set of durations observed for actions of class c. The instance based model was given by the corresponding empirical distribution function, so that $F_{\tau^*}(d \mid c) := n_c^{-1} \sum_{i=1}^{n_c} [d_{c,i} \leq d]$, where F_{τ^*} is the distribution function of τ^*. For the parametric models, an approach was chosen that would provide a trade-off between technical simplicity and flexibility. First, a lognormal model for the pooled observation was built, see Fig. 2 for a comparison with the actual durations and a kernel density estimate. Then those classes were determined whose means were significantly (at the .05 α level) different from the pooled mean by fitting an ANOVA model (analysis of variance). For these special classes as well as for the remaining pooled classes, the distribution giving the maximum likelihood was selected from a set of candidate distributions whose parameters were fitted to the observed class durations. The set of candidate distributions was Cauchy, exponential, gamma, geometric, lognormal, negative binomial, normal, Poisson, and Weibull.

The parametric models were expected to have an impact on performance for two reasons: (i) they might not provide a good fit to the empirical data, (ii) since they are continuous, they provide a greater number of possible durations, thereby increasing the branching factor and thus inference complexity.

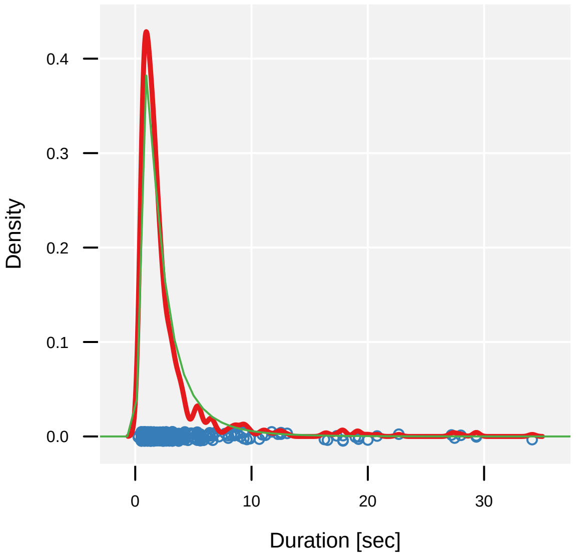

Figure 2. Kernel density estimate of pooled action durations vs. fitted lognormal density. Blue circles are the observed action durations. The red line is the kernel density estimate, the green line the fitted lognormal density.

S4.1.5 Action selection model

As primary goal-driven action selection feature, the goal distance feature f_δ as discussed in Appendix S2 was chosen. Goal distances were computed by an exhaustive process, computing the set of LTS states reachable from the initial state and then for all reachable states the shortest path to a goal state. For simplicity, unit costs were used for actions. This approach will become intractable with models of increasing state space complexity. In addition, it is not yet established that goal distance indeed best describes human action selection preferences. Approximate distance values therefore might achieve the same result, for a much lower computational cost. Two approximations were considered in this study:

- A goal distance heuristic f_h, that assigns heuristic distances to LTS states based on values of LTS state variables. The trial task's script consisting of 14 serial task steps was used to define a map from LTS state s to remaining script steps $h(s)$ based on its state variable values (see Tbl. S1 for this map). $h(s)$ then was used as distance value. (Most script tasks required several LTS actions for their realization.) The motivation of this heuristic is the idea that prior knowledge on the typical coarse-grained sequential structure of everyday activities should be easy to obtain [5,6]. Analyzing f_h thus should provide insight into the usefulness of such knowledge.
- A restricted goal distance feature $f_{\bar{\delta}}$. Here, only those LTS states were considered that are visited when using the annotations as exact observations. All other states discovered during inference received the nominal goal distance 100. Restricted goal distance $f_{\bar{\delta}}$ should give an upper limit to the gain achievable by a goal distance measure.

To gain insight on the effect of weight factors, each of these features was tested with the weight values $\lambda_i = -(2^k), k \in \{0, \ldots, 4\}$, using exponential probing. To limit experimental complexity, we refrained from evaluating interactions between features by only assigning one of $f_\delta, f_h, f_{\bar{\delta}}$ a non-zero weight.

In the special case $\lambda_\delta = \lambda_h = \lambda_{\bar{\delta}} = 0$ all applicable actions receive the same selection probability, resulting in uniform selection. (This *locally uniform* selection strategy is not the same as giving each

possible action *sequence* the same probability – such a *globally uniform* action selection model requires a considerable more complex feature computation.)

S4.2 iLTS model development

Building the inference LTS (iLTS, cf. Appendix S3) requires two steps: choosing an appropriate modeling language and implementing the model. The following two sections justify the choice and present the model development process used.

S4.2.1 Modeling language

As pure execution speed was not the primary concern of this study, Common Lisp was chosen as implementation language for the inference procedures. This allowed a very convenient way to represent actions in a PDDL / STRIPS like fashion as S-expressions, providing considerable latitude regarding the expressive power available for defining precondition terms and effect expressions, as essentially any valid Lisp expression could be used. For instance, the action of taking the food from the cutting board was defined by the following expression:

```
(:action (take food cutting_board)
     :precondition (and (eq ($ location self) ($ location cutting_board))
                        (eq ($ location food) 'cutting_board)
                        (> ($ available hands) 0))
     :effect (($ location food) 'hands
               ($ available hands) (- ($ available hands) 1)))
```

State variables are referred to by symbol lists, such as `($ location self)`. The precondition states that the protagonist (`self`) and the cutting board must be at the same location, the food must be on the cutting board, and the protagonist needs to have at least one free hand. The effect states that the location of the food will now be one of the protagonist's hand and that he has one less free hand available.

The state model was created by traversing all action definitions, collecting the state variable references, and compiling this to a Lisp `defstruct` containing one structure slot for each state variable. At the same time, action definitions were compiled to two pairs of Lisp functions, the first computing the precondition value, the second one for applying the effect to a state. State variable references were replaced by the corresponding slot access functions for the state `defstruct`; the value assignment in the effect was performed via `setf`. For efficiency considerations, only atomic values (symbols, characters, integers, and floating point numbers) were allowed as values for state variables. Therefore, states could be considered as words of constant length, allowing the use of tries as dictionaries for handling state sets, providing significantly faster access than hash tables in the Lisp environment [7] used in this study.

S4.2.2 Model development process

A two-stage process was used for iLTS model development. First a *feasible solution* $\mathcal{A}^*$ for the domain model (the set of action definitions) was constructed iteratively, and then this solution was refined to the final model $\mathcal{A}$ using local model modifications. For constructing the feasible solution, the domain expert used an iterative procedure for successively building action sets $\mathcal{A}_0, \mathcal{A}_1, \ldots, \mathcal{A}_S =: \mathcal{A}^*$ of increasing complexity.

Constructing the feasible solution begins by setting $\mathcal{A}_0 := \emptyset$. For the current action set $\mathcal{A}_s$, the model developer applies all action sequences obtained from the annotations in parallel and identifies the smallest t where the action $a_{i,t}$ of some dataset i fails at time t. For instance, the first action wash hands from the annotations of subject 1 (cf. Tbl. S3) is executed in the iLTS of $\mathcal{A}_0$, failing because this action is not included in $\mathcal{A}_0 = \emptyset$. If no action sequence fails, the feasible solution $\mathcal{A}^* := \mathcal{A}_s$ has been found.

Otherwise either (a) $a_{i,t}$ refers to an action not contained in $\mathcal{A}_s$ (as in the example), or (b) $a_{i,t}$ is in $\mathcal{A}_s$, but its precondition is not met in the current iLTS state after applying the preceding actions (for instance because `take` is executed twice, but was previously modeled to allow only one object in hand, cf. Tbl. S3, $t = 4$–8). In case (a), the model developer adds a new action schema with precondition and effect matching the informal semantics of the domain. In case (b), the model developer either relaxes the precondition or extends the effect of some other action such that the precondition of $a_{i,t}$ is met. It is the task of the domain expert to judge which of the refinement strategies to apply for reconciling a failure. The developer repeats the process with $\mathcal{A}_{s+1} := \mathcal{A}_s$ until a feasible solution has been found.

In the second step, the domain expert would refine $\mathcal{A}^*$ to the final action set $\mathcal{A}$ by adding preconditions in order to limit the number of possible plans. (For instance, in the case of the given trial setting, the protagonist would sit down at the table only after the food has been cooked). Note that this two stage process – consisting of the iterative construction of a feasible solution and then applying local search to refine the feasible solution towards a local optimum $\mathcal{A}$ – is similar to heuristic optimization strategies for solving integer programming problems.

References

1. Duda RO, Hart PE, Stork DG (2003) Pattern Classification. Wiley, second edition.

2. Boutilier C, Friedman N, Goldszmidt M, Koller D (1996) Context-specific independence in bayesian networks. In: Proceedings of the Twelfth International Conference on Uncertainty in Artificial Intelligence (UAI). San Francisco, CA, USA: Morgan Kaufmann Publishers Inc., pp. 115–123.

3. Milner B (1996) Inclusion of temporal information into features for speech recognition. In: Proceedings of the Fourth International Conference on Spoken Language. IEEE, volume 1, pp. 256–259.

4. Ostendorf M, Digalakis V, Kimball OA (1995) From hmms to segment models: A unified view of stochastic modeling for speech recognition. IEEE Transactions on Speech and Audio Processing 4: 360–378.

5. Rashidi P, Cook DJ (2013) COM: A method for mining and monitoring human activity patterns in home-based health monitoring systems. ACM Transactions of Intelligent Systems and Technology 4: 64:1–64:20.

6. Wyatt D, Philipose M, Choudhury T (2005) Unsupervised activity recognition using automatically mined common sense. In: Proceedings of the 20th national conference on Artificial Intelligence (AAAI). AAAI Press, pp. 21–27.

7. Clozure Associates. Clozure common lisp. URL `http://ccl.clozure.com/`. Accessed: 2014-03-26.
